# An image dataset related to automated macrophage detection in immunostained lymphoma tissue samples

**DOI:** 10.1093/gigascience/giaa016

**Published:** 2020-03-12

**Authors:** Marcus Wagner, Sarah Reinke, René Hänsel, Wolfram Klapper, Ulf-Dietrich Braumann

**Affiliations:** 1 Institute for Medical Informatics, Statistics and Epidemiology (IMISE), Leipzig University, Härtelstr. 16-18, D-04107 Leipzig, Germany; 2 Department of Pathology, Hematopathology Section and Lymph Node Registry, University of Kiel/University Hospital Schleswig-Holstein, Arnold-Heller-Str. 3, Haus 14, D-24105 Kiel, Germany; 3 Faculty of Engineering, Leipzig University of Applied Sciences (HTWK), P.O.B. 30 11 66, D-04251 Leipzig, Germany; 4 Fraunhofer Institute for Cell Therapy and Immunology (IZI), Perlickstr. 1, D-04103 Leipzig, Germany

**Keywords:** lymphoma, DLBCL, macrophage, multiple immunohistochemical staining, automated cell counting, ROF filtering, floating threshold, rule-based detection, image dataset

## Abstract

**Background:**

We present an image dataset related to automated segmentation and counting of macrophages in diffuse large B-cell lymphoma (DLBCL) tissue sections. For the classification of DLBCL subtypes, as well as for providing a prognosis of the clinical outcome, the analysis of the tumor microenvironment and, particularly, of the different types and functions of tumor-associated macrophages is indispensable. Until now, however, most information about macrophages has been obtained either in a completely indirect way by gene expression profiling or by manual counts in immunohistochemically (IHC) fluorescence-stained tissue samples while automated recognition of single IHC stained macrophages remains a difficult task. In an accompanying publication, a reliable approach to this problem has been established, and a large set of related images has been generated and analyzed.

**Results:**

Provided image data comprise (i) fluorescence microscopy images of 44 multiple immunohistostained DLBCL tumor subregions, captured at 4 channels corresponding to CD14, CD163, Pax5, and DAPI; (ii) ”cartoon-like” total variation–filtered versions of these images, generated by Rudin-Osher-Fatemi denoising; (iii) an automatically generated mask of the evaluation subregion, based on information from the DAPI channel; and (iv) automatically generated segmentation masks for macrophages (using information from CD14 and CD163 channels), B-cells (using information from Pax5 channel), and all cell nuclei (using information from DAPI channel).

**Conclusions:**

A large set of IHC stained DLBCL specimens is provided together with segmentation masks for different cell populations generated by a reference method for automated image analysis, thus featuring considerable reuse potential.

## Data Description

### Background

We present an image dataset generated as a part of an accompanying publication, which is concerned with method development and comparison for automated segmentation and counting of macrophages in diffuse large B-cell lymphoma (DLBCL) tissue sections [1]. DLBCL is an aggressive cancer disease that is characterized by a large heterogeneity of pathological, clinical, and biological features [2]. Therefore, a crucial step for the classification of DLBCL subtypes, as well as for providing a prognosis of the clinical outcome, is the analysis of the tumor microenvironment in terms of counts, local distributions, and functions of the different cell populations and, particularly, of the tumor-associated macrophages occuring there [3].

Until now, most information about macrophages has been obtained either by gene expression profiling [4] or by manual counts in immunohistochemically (IHC) stained tissue microarrays or high-power fields, thus either gathering information in a completely indirect way or accepting extreme subsampling rates [5]. A reliable approach for fully automated segmentation, identification, and counting of IHC stained macrophages within whole tissue slides has been addressed by Wagner et al. [1].

Our dataset contains monochrome fluorescence microscopy images of 44 DLBCL tissue samples wherein different macrophage populations (using antibodies against CD14 and CD163) and B-cells (using antibody against Pax5) as well as all cell nuclei (using 4´,6-diamidino-2-phenylindole [DAPI]) have been stained and imaged at different wavelengths. Furthermore, we supply processed images, comprising ”cartoon-like” total variation-filtered images (generated by Rudin-Osher-Fatemi [ROF] filtering), as well as results of the automated macrophage segmentation. For this publication, we completed these data by automated segmentation of B-cells and the cell nuclei.avoid headline numbering at all.

### Methods

#### Preparation and staining of DLBCL tissue

From the files of the Lymph Node Registry Kiel, 44 DLBCL biopsy specimens have been selected. For every specimen, from formalin-fixed paraffin-embedded tissue a slice of 2 µm thickness has been obtained. To detect specific macrophages and their relation to B-cells, a triple IHC staining was performed, using primary antibodies against CD14 (Cell Marque, Cat No. 114R-14, RRID:AB_2827391; 1:10), CD163 (Novus, Cat No. NB110-59935, RRID:AB_892323; 1:100), and Pax5 (Santa Cruz Biotechnology, Cat No. sc-1974, RRID:AB_2159678; 1:100) labeled with donkey anti-rabbit Alexa 488, donkey anti-mouse Alexa 555, and donkey anti-goat Alexa 647 (all from Invitrogen, Thermo Fisher Scientific, Waltham, MA, USA; 1:100) as secondary antibodies. Subsequently, the slices were incubated with DAPI (Invitrogen, Thermo Fisher Scientific, Waltham, MA, USA; 1:5,000) and cover-slipped with mounting medium. Use of tissue was in accordance with the guidelines of the internal review board of the Medical Faculty of the Christian-Albrechts-University Kiel, Germany (No. 447/10).

#### Selection of tumor subregions and image acquisition

Within every tissue sample, the tumor area was defined and marked by a pathologist on the basis of inspection of conventional hematoxylin-eosin (H&E) staining in a neighboring reference slice. Subsequently, within the IHC stained slice, a rectangular subregion of the tumor area was selected, taking care for acceptable tissue and staining quality. Maximum size of tumor subregions is 10 mm^2^.

Images of tumor subregions within the IHC stained slides were captured by Hamamatsu Nanozoomer 2.0 RS slide scanner (Hamamatsu Photonics, Ammersee, Germany) with 20× magnification at 4 wavelengths, resulting in single images for the CD14, CD163, Pax5, and DAPI channels, respectively, which were saved in .ndpi output format with default settings as used in clinical trial routine. Note that, at this point, moderate built-in compression by imaging device was accepted. Single-channel raw images were converted into .tif format without further compression and sliced into tiles of 1,000 × 1,000 pixel format (at right and lower border, the sizes may be smaller), using the software package ImageJ with the extension ndpitools [6]. The resulting monochrome images have been further converted from RGB into greyscale mode using the modulus of the RGB vector and finally saved in losslessly compressed .png format. We refer to them as images of type ”original.” Note that image acquisition and tiling have been performed in such a way that no spatial misalignment between the scans at the different wavelengths occurred. Pixel size is 0.45 × 0.45 µm^2^ in all images.

#### Image processing

For every tile, the segmentation method from Wagner et al. [1] has been applied to the CD14, CD163, Pax5, and DAPI channel images, resulting in ROF-filtered images (saved as type ”cartoon”); a mask for the evaluation subregion within the tile, indicating the presence of tissue at all, as inferred from DAPI channel information (saved as type ”evalmask”); and segmentations of macrophages within the CD14 and CD163 channels (saved as type "segment"). Owing to the large inhomogeneity of IHC staining, even across a single target macrophage, we provide 2 further masks containing the convex hulls of the segmented features instead of the features themselves (saved as type "convhull"). The segmentation masks for double-stained macrophages are saved as type “multiple”. For a general description of the ROF filter–based segmentation method, see Wagner et al. [1]. Here, we describe in more detail the generation of segmentations for the Pax5 and DAPI channels, which are new in this article.

Let us recall the notation from [1] where the indices *i* and *j* count the current intensity threshold and the features to be inspected at this stage, *s*(*F*_*j*_), *c*(*F*_*j*_), and *r*(*F*_*j*_), denote the size of a feature *F*_*j*_ itself, the size of its convex hull, and the ratio of the principal axes’ lengths of the smallest ellipse covering the feature, respectively. Meanwhile, *s*_min_, *s*_max_, *c*_max_, and *r*_max_ denote the minimal and maximal feature size (in pixels), the maximal area excess of the convex hull (in percent), and the maximal ratio of axes, respectively.

To obtain a segmentation of the DAPI channel, the ROF-filtered image has been further subjected to a local Narendra-Fitch contrast enhancement [7]

(1) $$\begin{eqnarray*}
p(k,l)_{\mathrm{enhanced}}=m(k,l) + {c\over \sigma(k,l)}\cdot[p(k,l)_{\mathrm{original}} - m(k,l)],
\end{eqnarray*}$$

where *c* > 0 is a weight parameter and *m*(*k*, *l*), σ(*k*, *l*) denote the mean and standard deviation of the intensities within a subregion centered at the pixel *p*(*k*, *l*)_original_, respectively. We used *c* = 0.75 and a square subregion of 11 × 11 pixels size. Then, in a first run, Steps 3–10 of the ROF filter–based segmentation have been applied, using the bounds *s*_min_ = 60 and *s*_max_ = 119 for the feature size but modifying geometrical Rule 3) for feature classification from [1] as follows: If *s*_min_ ≤ *s*(*F*) ≤ *s*_max_, then test whether the feature satisfies both of the Criteria 3b) *r*(*F*_*j*_) ≤ *r*_max_ (the feature is not too elongated) and 3d) *c*(*F*_*j*_)/*s*(*F*_*j*_) ≤ 1 + *c*_max_/100 (the deviation from circular shape is bounded from above). If yes, save the feature *F*_*j*_ into the output mask, interpreting it as a cell nucleus, and mask it in *I*^(3)^(*i*). If not, then neglect the feature and mask it in *I*^(3)^(*i*) as well. Here, we used the parameter values *r*_max_ = 2.5 and *c*_max_ = 150. In a second run, Steps 3–10 of the ROF filter–based segmentation have been repeated with the parameter settings *s*_min_ = 120 and *s*_max_ = 180, using again the described modification of Rule 3) but saving only those features into the output mask that are completely disjoint to the output of the first run. Finally, the results of both runs have been combined into a single mask (saved as type "segment"). Within a further result mask of type convhull, the convex hulls of the detected features have been stored.

For the segmentation of the B-cells, the ROF-filtered image of the Pax5 channel has been subjected to a moderate Narendra-Fitch contrast enhancement as well, using the parameter *c* = 0.1 and a square subregion of 15 × 15 pixels size. To the result, Steps 3–10 of the ROF filter-based segmentation have been applied, using the bounds *s*_min_ = 80 and *s*_max_ = 159 as well as the described modification of Rule 3) with parameters *r*_max_ = 2.5 and *c*_max_ = 150 but saving into the output mask (of type segment) only features whose intersection with the convex hull of some cell nucleus, as obtained in the segmentation of the DAPI channel, is nonempty. Thus, numerous artifacts appearing in the Pax5 staining will be excluded. Again, the convex hulls of the dectected features have been stored within a further mask of type convhull.

#### BLC2 scoring

For all specimens, a BCL2 score is available (see Table 2). It is based on BCL2 staining for tissue slides obtained from the same biopsy specimens as before but not necessarily adjacent to the slides used for the generation of the image data presented here. Staining was microscopically examined and semi-quantitatively scored by an experienced pathologist. Each stained slide was evaluated for the percentage of stained tumor cells by visual estimation in a representative tumor area. The estimated value was graded into the following scores: 0, all cells negative; 1, ≤25% positive cells; 2, 26–50% positive cells; 3, 51–75% positive cells; 4, >75% positive cells.

### Dataset structure

**Figure 1:**
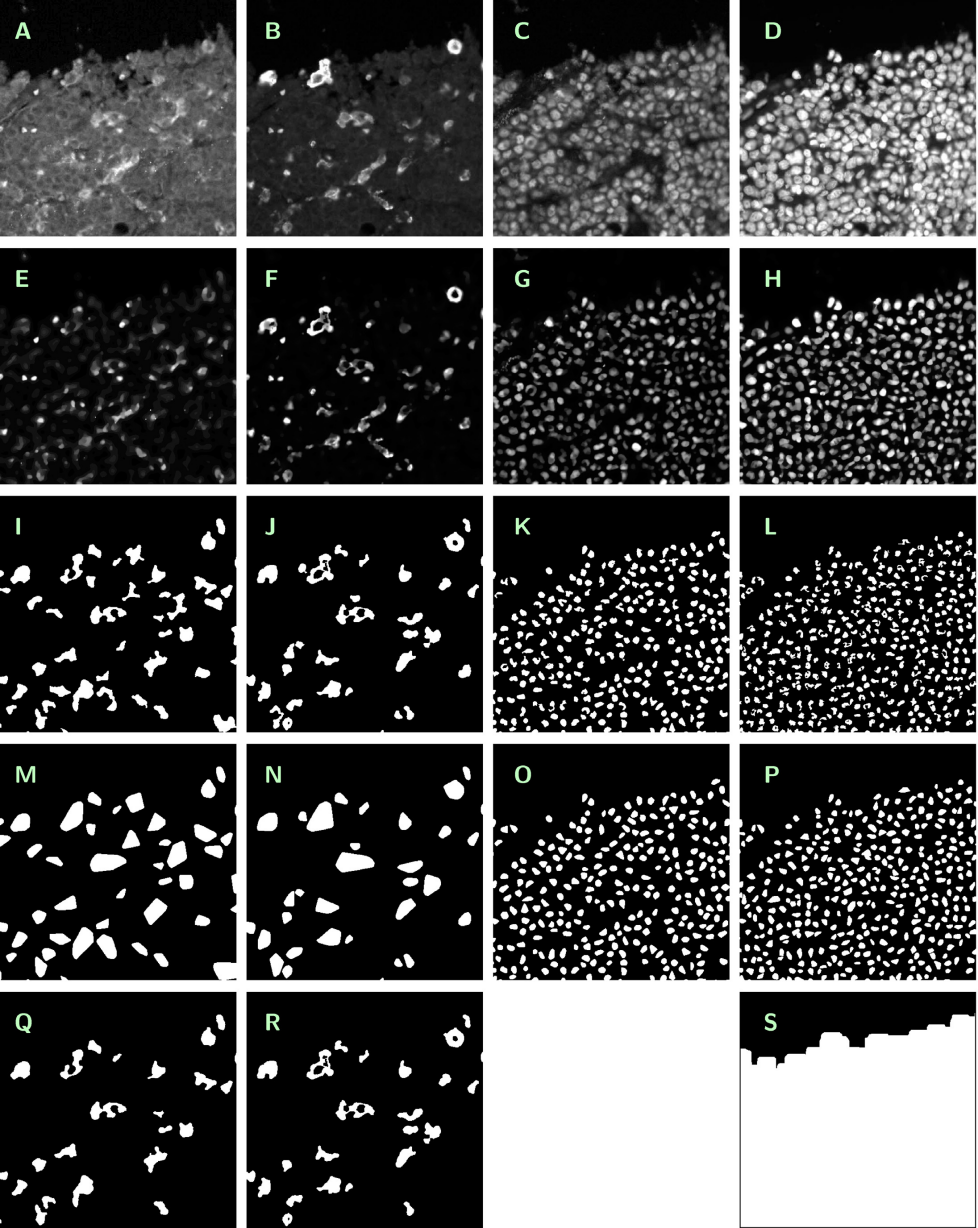
Summary of images available at a given tile (cutouts of 500 × 500 pixels size). Contrast enhanced in A by a factor of 3.5, in E by a factor of 7, and in F–H by a factor of 2. *Originals (A–D)*. A: specimen_02_tile_01_06_channel_CD14_type_original_mode_gs.png, B: specimen_02_tile_01_06_channel_CD163_type_original_mode_gs.png, C: specimen_02_tile_01_06_channel_Pax5_type_original_mode_gs.png, D: specimen_02_tile_01_06_channel_DAPI_type_original_mode_gs.png. *Cartoons (E–H)*. E: specimen_02_tile_01_06_channel_CD14_type_cartoon_mode_gs.png, F: specimen_02_tile_01_06_channel_CD163_type_cartoon_mode_gs.png, G: specimen_02_tile_01_06_channel_Pax5_type_cartoon_mode_gs.png, H: specimen_02_tile_01_06_channel_DAPI_type_cartoon_mode_gs.png, *Segmentations (I–L)*. I: specimen_02_tile_01_06_channel_CD14_type_segment_mode_bw.png, J: specimen_02_tile_01_06_channel_CD163_type_segment_mode_bw.png, K: specimen_02_tile_01_06_channel_Pax5_type_segment_mode_bw.png, L: specimen_02_tile_01_06_channel_DAPI_type_segment_mode_bw.png,*Convex hulls (M–P)*. M: specimen_02_tile_01_06_channel_CD14_type_convhull_mode_bw.png, N: specimen_02_tile_01_06_channel_CD163_type_convhull_mode_bw.png, O: specimen_02_tile_01_06_channel_Pax5_type_convhull_mode_bw.png, P: specimen_02_tile_01_06_channel_DAPI_type_convhull_mode_bw.png,*Various (Q–S)*. Q: specimen_02_tile_01_06_channel_CD14_type_multiple_mode_bw.png, R: specimen_02_tile_01_06_channel_CD163_type_multiple_mode_bw.png, S: specimen_02_tile_01_06_channel_DAPI_type_evalmask_mode_bw.png.

Image data are organized by tissue specimens (top-level folders) and tiles (second-level folders), the latter ordered by position. Top-level folders are named specimen_01, ... , specimen_44; second-level folders are named, e.g., specimen_01_tile_01_01, ... , specimen_01_tile_09_08. Within each second-level folder, 19 image files in greyscale (gs) or black-and-white (bw) mode are stored in losslessly compressed .png format with 8-bit or 1-bit depth, respectively. Table 1 and Fig. 1 summarize the different images available at a given tile. The filenames are built as specimen_xx_tile_yy_zz_channel_[CD14, CD163, Pax5, DAPI]_type_[original, cartoon, segment, convhull, multiple, evalmask]_mode_[gs, bw].png. The size of losslessly compressed .png image files has been minimized by application of the OptiPNG routine [8]. Moreover, a logfile named specimen_xx_tile_yy_zz__logfile.txt is provided, containing detailed information about procedures, parameters, and results of automated segmentation.

**Table 1.** Image files available within a given second-level folder

Channel	Description	Type	Mode
CD14	CD14 staining, original single-channel image	original	gs
	ROF-filtered image derived from original	cartoon	gs
	Mask highlighting the segmented macrophages	segment	bw
	Mask highlighting the convex hulls of the segmented macrophages	convhull	bw
	Mask highlighting the segmented macrophages bearing CD163 staining as well	multiple	bw
CD163	CD163 staining, original single-channel image	original	gs
	ROF-filtered image derived from original	cartoon	gs
	Mask highlighting the segmented macrophages	segment	bw
	Mask highlighting the convex hulls of the segmented macrophages	convhull	bw
	Mask highlighting the segmented macrophages bearing CD14 staining as well	multiple	bw
Pax5	Pax5 staining, original single-channel image	original	gs
	ROF-filtered image derived from original	cartoon	gs
	Mask highlighting the segmented B-cells	segment	bw
	Mask highlighting the convex hulls of the segmented B-cells	convhull	bw
DAPI	DAPI staining, original single-channel image	original	gs
	ROF-filtered image derived from original	cartoon	gs
	Mask representing the evaluation subregion	evalmask	bw
	Mask highlighting the segmented cell nuclei	segment	bw
	Mask highlighting the convex hulls of the segmented cell nuclei	convhull	bw

### Reuse potential

Although there is a vast number of publications concerned with the composition of the tumor microenvironment in various types of lymphoma disease, image datasets of IHC stained cancer tissue are rarely publicly accessible if at all (cf. the discussion in Kostopoulos et al. [9]). Most data generated for the purpose of such analyses are not findable or not even accessible. For example, the Genomic Data Commons Data Portal of the National Cancer Institute [10,11] currently lists only 48 cases of mature B-cell lymphoma with an image of an H&E-stained slide available, while IHC stainings are completely missing. In this situation, the image dataset presented in this article constitutes a document of interest in itself.

We outline the most important options for further use of the dataset. First, it allows for a detailed morphometrical investigation of the imaged macrophages and B-cells with respect to the distribution of geometrical parameters such as size, diameter, and perimeter, as well as to overall shape patterns. Second, the data may be used for validation, calibration, and comparison of cell segmentation methods (manual, automated) and related software packages, making available a large reference dataset together with the output of a reference method as described in [1]. Note that, for these purposes, it is particularly adequate to use data featuring a routine quality level. Third, the original images as well as the segmentations presented here could be used for the generation of a sufficiently large training set for automated macrophage detection by machine learning methods. Fourth, the data may be used for study of co-localization and clustering of macrophages and B-cells within lymphoma tissue and cancer microenvironment, using appropriate methods of point-pattern statistics [12,13]. Finally, the dataset enables a closer study of the double-stained macrophage subpopulation. To facilitate a possible further processing of the obtained features (e.g., extraction of barycenters, replacement of the features by equally sized circles or squares), not only the masks for the segmented features themselves but as well those for their convex hulls are provided.

**Figure 2:**
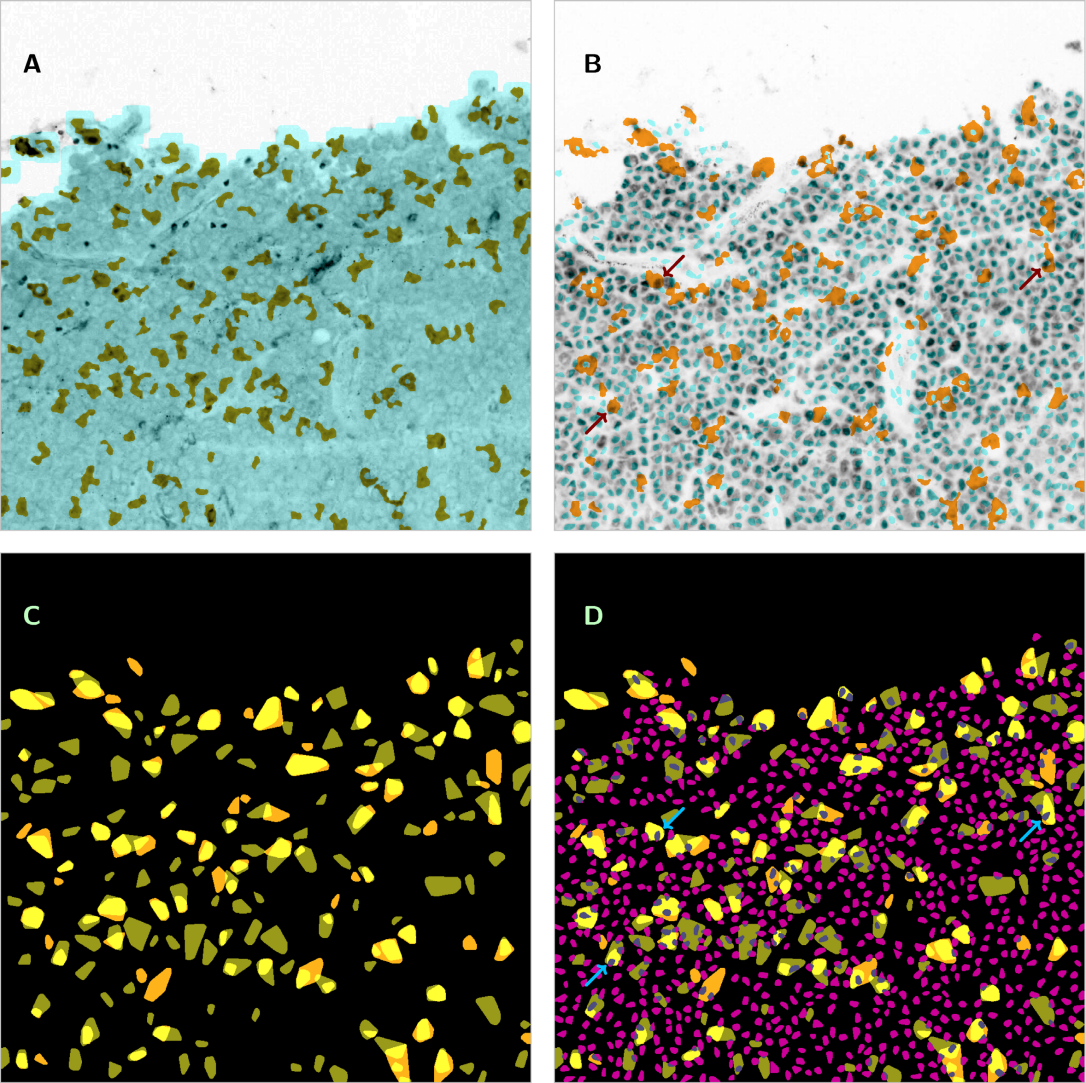
Examples of combined information from several images, based on specimen_02_tile_01_06_... Image size is 1,000 × 1,000 pixels (450 × 450 μm^2^). A: Original image at CD14 channel (greyscale, contrast enhanced by a factor of 3.5, inverted) (channel_CD14_type_original_mode_gs.png) with superimposition of the mask of the evaluation subregion, as obtained from the DAPI channel (light blue) (channel_DAPI_type_evalmask_mode_bw.png) and the segmentation of the CD14-stained macrophages (olive green) (channel_CD14_type_segment_mode_bw.png). B: The same tile as imaged at the Pax5 channel (greyscale, inverted) (channel_Pax5_type_original_mode_gs.png) with superimposition of the cell nuclei segmentation from the DAPI channel (light blue, convex hulls) (channel_DAPI_type_convhull_mode_bw.png) and the segmentation of the CD163-stained macrophages (dark yellow) (channel_CD163_type_segment_mode_bw.png). Examples of B-cells positioned inside macrophages indicated by arrows. C: Combination of both macrophage segmentations (olive green or dark yellow, convex hulls) for the same tile in order to reveal double-stained parts (light yellow) ( channel_CD14_type_convhull_mode_bw.png / channel_CD163_type_convhull_mode_bw.png). D: Segmentation of B-cells from the Pax5 channel (magenta and grey, convex hulls) (channel_Pax5_type_convhull_mode_bw.png) superimposed to Fig. 2C. Examples of B-cells positioned inside macrophages indicated by arrows (the same cells as in Fig. 2B).

To illustrate the described reuse potential, we include a set of composite figures, each combining information from several separate images. Fig. 2A shows an original image at CD14 channel (greyscale, original contrast-enhanced by a factor of 3.5 and inverted) with superimposition of the mask of the evaluation subregion, as obtained from the DAPI channel (light blue), and the segmentation of the CD14-stained macrophages (olive green). Fig. 2B shows the same tile as imaged at the Pax5 channel (greyscale, original inverted) with superimposition of the cell nuclei segmentation from the DAPI channel (light blue, convex hulls) and the segmentation of the CD163-stained macrophages (dark yellow). In Fig. 2C, for the same tile, both macrophage segmentations (olive green or dark yellow, convex hulls) are combined to reveal double-stained parts (light yellow). In Fig. 2D, we superimposed to Fig. 2C the segmentation of B-cells from the Pax5 channel (magenta and grey, convex hulls). Observe that in Figs 2B and D, some B-cells are positioned inside of macrophages, indicating that they are engulfed by the macrophages for phagocytosis (examples marked by arrows). It is obvious that co-localization and clustering patterns as empirically noticeable here must be investigated on a sound base of statistical methodology.

To improve reusability, BLC2 scores for the biopsy specimens are provided.

**Table 2.** BLC2 scores

Score	Specimen No.
0	03, 05, 18, 32, 37, 43, 44
1	--
2	36
3	28
4	01, 02, 04, 06–17, 19–27, 29–31, 33–35, 38–42

## Availability of Supporting Data and Materials

All image data are made publicly accessible under CC0 1.0 license at the Leipzig Health Atlas (LHA) repository [14] and can be reached from the website [15]. Each top-level folder can be downloaded as .zip file and bears a separate identifier, e.g., https://health-atlas.de/lha/7YXMMFNPDG-0 within the repository (see Table 3). Two folders with total size >1 GB (Nos. 04 and 44) have been split into a pair of files. Snapshots of the datasets are available in the *GigaScience* GigaDB repository as well [16].

**Table 3.** Datasets available at the Leipzig Health Atlas

Name	Size (MB)	Identifier
specimen_01.zip	161	7YXMMFNPDG-0
specimen_02.zip	142	7YXXYUTPYN-9
specimen_03.zip	121	7YXY2MUWDK-3
specimen_04a.zip	630	7YXYECRXQM-0
specimen_04b.zip	709	7YXYY60JX7-9
specimen_05.zip	653	7YY08G00A0-4
specimen_06.zip	168	7YY0NHJXF8-2
specimen_07.zip	409	7YY0X073KU-7
specimen_08.zip	396	7YY146X8HE-4
specimen_09.zip	283	7YY19AWR7C-8
specimen_10.zip	368	8004FF6QR6-5
specimen_11.zip	708	8004RQHWHX-6
specimen_12.zip	360	800516PXMC-9
specimen_13.zip	150	8005NDPDNX-6
specimen_14.zip	283	8005QXY7QG-0
specimen_15.zip	124	8005TY4388-4
specimen_16.zip	146	8005X2U355-5
specimen_17.zip	364	80062H7C7J-4
specimen_18.zip	164	800HNVWTJX-5
specimen_19.zip	105	800HR9GPJE-5
specimen_20.zip	418	800J5AN4V1-7
specimen_21.zip	431	800JDAJXHV-6
specimen_22.zip	465	802X2RAVTV-8
specimen_23.zip	333	802X8VYQ27-0
specimen_24.zip	462	802XR67DWU-2
specimen_25.zip	749	802Y1JFKPQ-6
specimen_26.zip	635	803AHC5EAH-5
specimen_27.zip	137	803AHW6TD9-4
specimen_28.zip	225	803AU2NYKJ-8
specimen_29.zip	549	803C11PMP7-3
specimen_30.zip	334	803C4Q94NP-5
specimen_31.zip	217	803C809ERJ-6
specimen_32.zip	293	803CFU4J96-9
specimen_33.zip	330	803CJR62YA-8
specimen_34.zip	474	803NYKM0PY-9
specimen_35.zip	286	803PH07HQT-2
specimen_36.zip	225	803PJKT2JG-7
specimen_37.zip	563	803PKWG9XG-9
specimen_38.zip	524	803PPV4R44-8
specimen_39.zip	879	8044GTGCPG-1
specimen_40.zip	382	8044J0U5JC-0
specimen_41.zip	421	804GHX9A2E-8
specimen_42.zip	114	804GJF4HQ4-8
specimen_43.zip	596	804GY21PMN-9
specimen_44a.zip	506	804H6EM8W2-5
specimen_44b.zip	452	804H7C4T1P-0

## Abbreviations

DAPI: 4´,6-diamidino-2-phenylindole; DLBCL: diffuse large B-cell lymphoma; H&E: hematoxylin-eosin; IHC: immunohistochemical(ly); LHA: Leipzig Health Atlas; ROF: Rudin-Osher-Fatemi.

## Ethical approval

Tissue usage is covered by statement No. 447/10 of the internal review board of the Medical Faculty of the Christian-Albrechts-University Kiel, Germany.

## Competing interests

The authors declare that they have no competing interests.

## Supplementary Material

giaa016_GIGA-D-19-00311_Original_Submission

giaa016_GIGA-D-19-00311_Revision_1

giaa016_GIGA-D-19-00311_Revision_2

giaa016_Response_to_Reviewer_Comments_Original_Submission

giaa016_Response_to_Reviewer_Comments_Revision_1

giaa016_Reviewer_1_Report_Original_Submission Chris Armit -- 9/23/2019 Reviewed

giaa016_Reviewer_1_Report_Revision_1 Chris Armit -- 1/16/2020 Reviewed

giaa016_Reviewer_2_Report_Original_Submission Guy M Hagen, PHD -- 9/26/2019 Reviewed

giaa016_Reviewer_2_Report_Revision_1 Guy M Hagen, PHD -- 1/13/2020 Reviewed
