# Supplementary material for: An image dataset related to automated macrophage detection in immunostained lymphoma tissue samples
Source: Gigascience. 2020 Mar 12;9(3):giaa016. doi: 10.1093/gigascience/giaa016 (PMC7066390; doi:10.1093/gigascience/giaa016)
Supplement: giaa016_GIGA-D-19-00311_Original_Submission [file giaa016_giga-d-19-00311_original_submission.pdf]

# GigaScience


## An image dataset related to automated macrophage detection in immunostained lymphoma tissue samples

--Manuscript Draft--

| | |
|---|---|
| **Manuscript Number:** | GIGA-D-19-00311 |
| **Full Title:** | An image dataset related to automated macrophage detection in immunostained lymphoma tissue samples |
| **Article Type:** | Data Note |
| **Funding Information:** | BMBF (031A428D) — Dr. Marcus Wagner, Mr. René Hänsel<br>BMBF (031A428C) — Dr. Sarah Reinke |
| **Abstract:** | Background: We present an image dataset related to automated segmentation and counting of macrophages in diffuse large B-cell lymphoma {(DLBCL)} tissue sections. For the classification of DLBCL subtypes as well as for as for providing a prognosis of the clinical outcome, the analysis of the tumor microenvironment and, particularly, of the different types and functions of tumor-associated macrophages, is indispensable. Until now, however, most information about macrophages is obtained either in a completely indirect way by gene expression profiling or by manual counts in immunohistochemically (IHC) fluorescence stained tissue samples while automated recognition of single IHC stained macrophages remains a difficult task. In an accompanying publication, a reliable approach to this problem has been established, and a large set of related images has been generated and analyzed.<br><br>Results: Provided image data comprise a) fluorescence microscopy images of 44 multiple immunohistostained DLBCL tumor subregions, captured at four channels corresponding to CD14, CD163, Pax5 and DAPI; b) cartoon-filtered versions of these images, generated by Rudin-Osher-Fatemi (ROF) denoising; c) an automatically generated mask of the evaluation subregion, based on information from the DAPI channel, and d) automatically generated segmentation masks for macrophages, B-cells and the total of cell nuclei, using information from CD14, CD163, Pax5 and DAPI channels, respectively.<br><br>Conclusions: A large set of IHC stained DLBCL specimens is provided together with segmentation masks for different cell populations generated by a reference method for automated image analysis, thus featuring considerable reuse potential. |
| **Corresponding Author:** | Marcus Wagner<br>Universitat Leipzig<br>Leipzig, GERMANY |
| **Corresponding Author Secondary Information:** | |
| **Corresponding Author's Institution:** | Universitat Leipzig |
| **Corresponding Author's Secondary Institution:** | |
| **First Author:** | Marcus Wagner |
| **First Author Secondary Information:** | |
| **Order of Authors:** | Marcus Wagner<br>Sarah Reinke<br>René Hänsel<br>Wolfram Klapper<br>Ulf-Dietrich Braumann |


*Powered by Editorial Manager® and ProduXion Manager® from Aries Systems Corporation*

| Order of Authors Secondary Information: | |
|---|---|
| Additional Information: | |
| Question | Response |
| Are you submitting this manuscript to a special series or article collection? | No |
| **Experimental design and statistics**<br><br>Full details of the experimental design and statistical methods used should be given in the Methods section, as detailed in our Minimum Standards Reporting Checklist. Information essential to interpreting the data presented should be made available in the figure legends.<br><br>Have you included all the information requested in your manuscript? | Yes |
| **Resources**<br><br>A description of all resources used, including antibodies, cell lines, animals and software tools, with enough information to allow them to be uniquely identified, should be included in the Methods section. Authors are strongly encouraged to cite Research Resource Identifiers (RRIDs) for antibodies, model organisms and tools, where possible.<br><br>Have you included the information requested as detailed in our Minimum Standards Reporting Checklist? | Yes |
| **Availability of data and materials**<br><br>All datasets and code on which the conclusions of the paper rely must be either included in your submission or deposited in publicly available repositories (where available and ethically appropriate), referencing such data using a unique identifier in the references and in the "Availability of Data and Materials" section of your manuscript. | Yes |

| | |
|---|---|
| Have you have met the above requirement as detailed in our Minimum Standards Reporting Checklist? | |

Manuscript
Click here to download Manuscript yh_004.pdf

*GigaScience*, 2019, 1–7

doi: xx.xxxx/xxxx
Manuscript in Preparation
Data Note

DATA NOTE

# An image dataset related to automated macrophage detection in immunostained lymphoma tissue samples

Marcus Wagner[1,*,†], Sarah Reinke[2,†], René Hänsel[1], Wolfram Klapper[2] and Ulf-Dietrich Braumann[3]

[1]Institute for Medical Informatics, Statistics and Epidemiology (IMISE), University of Leipzig, Härtelstr. 16-18, D-04107 Leipzig, Germany and [2]Department of Pathology, Hematopathology Section and Lymph Node Registry, University of Kiel / University Hospital Schleswig-Holstein, Arnold-Heller-Str. 3, Haus 14, D-24105 Kiel, Germany and [3]Faculty of Electrical Engineering and Information Technology, Leipzig University of Applied Sciences (HTWK), P. O. B. 30 11 66, D-04251 Leipzig, Germany / Fraunhofer Institute for Cell Therapy and Immunology (IZI), Perlickstr. 1, D-04103 Leipzig, Germany

[*] Correspondence address. Institute for Medical Informatics, Statistics and Epidemiology (IMISE), University of Leipzig, Härtelstr. 16-18, D-04107 Leipzig, Germany. E-mail: marcus.wagner@imise.uni-leipzig.de
[†] Contributed equally.

## Abstract

**Background:** We present an image dataset related to automated segmentation and counting of macrophages in diffuse large B-cell lymphoma (DLBCL) tissue sections. For the classification of DLBCL subtypes as well as for as for providing a prognosis of the clinical outcome, the analysis of the tumor microenvironment and, particularly, of the different types and functions of tumor-associated macrophages, is indispensable. Until now, however, most information about macrophages is obtained either in a completely indirect way by gene expression profiling or by manual counts in immunohistochemically (IHC) fluorescence stained tissue samples while automated recognition of single IHC stained macrophages remains a difficult task. In an accompanying publication, a reliable approach to this problem has been established, and a large set of related images has been generated and analyzed.
**Results:** Provided image data comprise a) fluorescence microscopy images of 44 multiple immunohistostained DLBCL tumor subregions, captured at four channels corresponding to CD14, CD163, Pax5 and DAPI; b) cartoon-filtered versions of these images, generated by Rudin-Osher-Fatemi (ROF) denoising; c) an automatically generated mask of the evaluation subregion, based on information from the DAPI channel, and d) automatically generated segmentation masks for macrophages, B-cells and the total of cell nuclei, using information from CD14, CD163, Pax5 and DAPI channels, respectively.
**Conclusions:** A large set of IHC stained DLBCL specimens is provided together with segmentation masks for different cell populations generated by a reference method for automated image analysis, thus featuring considerable reuse potential.

**Key words**: lymphoma, DLBCL, macrophage, multiple immunohistochemical staining, automated cell counting, ROF filtering, floating threshold, rule-based detection, image dataset

**Compiled on:** September 3, 2019.
Draft manuscript prepared by the author.

# Data Description

## Context

We present an image dataset generated as a part of an accompanying publication, which is concerned with method development and comparison for automated segmentation and counting of macrophages in diffuse large B-cell lymphoma (DLBCL) tissue sections [1]. DLBCL is an aggressive cancer disease which is characterized by a large heterogeneity of pathological, clinical and biological features [2]. Therefore, a crucial step for the classification of DLBCL subtypes as well as for providing a prognosis of the clinical outcome is the analysis of the tumor microenvironment in terms of counts, local distributions and functions of the different cell populations and, particularly, of the tumor-associated macrophages occuring there [3].

Until now, most information about macrophages is obtained either by gene expression profiling [4] or by manual counts in immunohistochemically (IHC) stained tissue microarrays resp. high-power fields, thus either gathering information in a completely indirect way or accepting extreme subsampling rates [5]. A reliable approach for fully automated segmentation, identification and counting of IHC stained macrophages within whole tissue slides has been addressed in [1].

Our dataset contains monochrome fluorescence microscopy images of 44 DLBCL tissue samples wherein different macrophage populations (using antibodies against CD14 and CD163) and B-cells (using antibody against Pax5) as well as the total of cell nuclei (using DAPI) have been stained and imaged at different wavelengths. Further, we supply processed images, comprising cartoons (generated by Rudin-Osher-Fatemi filtering) as well as results of the automated macrophage segmentation. For this publication, we completed these data by automated segmentation of B-cells and the total of cell nuclei.

# Methods

*a) Preparation and staining of DLBCL tissue.* From the files of the Lymph Node Registry Kiel, 44 DLBCL biopsy specimens have been selected. For every specimen, from formalin-fixed paraffin-embedded tissue a slice of 2 $\mu$m thickness has been obtained. In order to detect specific macrophages and its relation to B-cells, a triple IHC staining has been done, using primary antibodies against CD14 (Clone EPR3653; Cell Marque, Rocklin, CA, USA; 1:10), CD163 (Clone 10D6; Novocastra, Leica Biosystems, Wetzlar, Germany; 1:100) and Pax5 (polyclonal; Santa Cruz Biotechnology, Heidelberg, Germany; 1:100) labelled with donkey anti rabbit Alexa 488, donkey anti mouse Alexa 555 and donkey anti goat Alexa 647 (all from Invitrogen, Thermo Fisher Scientific, Waltham, MA, USA; 1:100) as secondary antibodies. Subsequently, the slices have been incubated with DAPI (Invitrogen, Thermo Fisher Scientific, Waltham, MA, USA; 1:5000) and cover-slipped with mounting medium. Use of tissue was in accordance with the guidelines of the internal review board of the Medical Faculty of the Christian-Albrechts-University Kiel, Germany (No. 447/10).

*b) Selection of tumor subregions and image acquisition.* Within every tissue sample, the tumor area was defined and marked by a pathologist based on inspection of conventional Haematoxylin-Eosin (HE) staining in a neighboring reference slice. Subsequently, within the IHC stained slice, a rectangular subregion of the tumor area has been selected, taking care for acceptable tissue and staining quality. Maximum size of tumor subregions is 10 $\text{mm}^2$.

Images of tumor subregions within the IHC stained slides have been captured by Hamamatsu Nanozoomer 2.0 RS slide scanner (Hamamatsu Photonics, Ammersee, Germany) with $20\times$ magnification at four wavelengths, resulting in single images for the CD14, CD163, Pax5 and DAPI channels, respectively. Pixel size is $0.45 \times 0.45\,\mu$m in all images. Single-channel raw images have been converted into uncompressed `.tif` format and sliced into tiles of $1000 \times 1000$ px format (at right and lower border, the sizes may be smaller), using the software package ImageJ with the extension ndpitools [6]. The resulting monochrome images have been further converted from RGB into greyscale mode using the modulus of the RGB vector and finally saved in losslessly compressed `.png` format. We refer to them as to images of type "original". Let us remark that image acquisition and tiling have been performed in such a way that no spatial misalignment between the scans at the different wavelengths occurred.

*c) Image processing.* For every tile, the segmentation method from [1] has been applied to the CD14, CD163, Pax5 and DAPI channel images, resulting in ROF-filtered cartoons of the images (saved as type "cartoon"), a mask for the evaluation subregion within the tile, indicating the presence of tissue at all, as inferred from DAPI channel information (saved as type "evalmask"), and segmentations of macrophages within the CD14 and CD163 channels (saved as type "segment"). Due to the large inhomogeneity of IHC staining, even across a single target macrophage, we provide two further masks containing the convex hulls of the segmented features instead of the features themselves (saved as type "convhull"). The segmentation masks for double-stained macrophages are saved as type "multiple". For a general description of the ROF filter based segmentation method, we refer to [1]. Here, we describe in more detail the generation of segmentations for the Pax5 and DAPI channels, which are new in this paper.

Let us recall the notation from [1] where the indices $i$ and $j$ count the current intensity threshold and the features to be inspected at this stage, $s(F_j)$, $c(F_j)$ and $r(F_j)$ denote the size of a feature $F_j$ itself, the size of its convex hull and the ratio of the principal axes' lengths of the smallest ellipse covering the feature, respectively. $s_{min}$, $s_{max}$, $c_{max}$ and $r_{max}$ denote the minimal and maximal feature size (in px), the maximal area excess of the convex hull (in percent) and the maximal ratio of axes, respectively.

In order to obtain a segmentation of the DAPI channel, the ROF-filtered cartoon has been subjected to a local Narendra-Fitch contrast enhancement [7]

$$p(k,l)_{enhanced} = m(k,l) + \frac{c}{\sigma(k,l)} \cdot \Big( p(k,l)_{original} - m(k,l) \Big) \quad (1)$$

where $c > 0$ is a weight parameter and $m(k,l)$, $\sigma(k,l)$ denote the mean and standard deviation of the intensities within a subregion centered at the pixel $p(k,l)_{original}$, respectively. We used $c = 0.75$ and a square subregion of $11 \times 11$ px size. Then, in a first run, Steps 3 – 10 of the ROF filter based segmentation have been applied, using the bounds $s_{min} = 60$ and $s_{max} = 119$ for the feature size but modifying geometrical rule No. 3) for feature classification from [1] as follows: If $s_{min} \leq s(F) \leq s_{max}$ then test whether the feature satisfies both of the criteria 3b) $r(F_j) \leq r_{max}$ (the feature is not too elongated) and 3d) $c(F_j)/s(F_j) \leq 1 + c_{max}/100$ (the deviation from circular shape is bounded from above). If yes, save the feature $F_j$ into the output mask, interpreting it as a cell nucleus, and mask it in $I^{(3)}(i)$. If not then neglect the feature and mask it in $I^{(3)}(i)$ as well. Here, we used the parameter values $r_{max} = 2.5$ and $c_{max} = 150$. In a second run, Steps 3 – 10 of the ROF filter based segmentation have been repeated with the parameter settings $s_{min} = 120$ and $s_{max} = 180$, using again the described modification of rule No. 3) but saving only those features into the output mask which are completely disjoint to the output of the first run. Finally, the

results of both runs have been combined into a single mask (saved as type "segment"). Within a further result mask of type "convhull", the convex hulls of the detected features have been stored.

For the segmentation of the B-cells, the ROF-filtered cartoon of the Pax5 channel has been subjected to a moderate Narendra-Fitch contrast enhancement as well, using the parameter $c = 0.1$ and a square subregion of $15 \times 15$ px size. To the result, Steps 3 – 10 of the ROF filter based segmentation have been applied, using the bounds $s_{min} = 80$ and $s_{max} = 159$ as well as the described modification of rule No. 3) with parameters $r_{max} = 2.5$ and $c_{max} = 150$ but saving into the output mask (of type "segment") only features which intersection with the convex hull of some cell nucleus, as obtained in the segmentation of the DAPI channel, is nonempty. Thus, numerous artifacts appearing in the Pax5 staining will be excluded. Again, the convex hulls of the dectected features have been stored within a further mask of type "convhull".

**Table 1.** Image files available within a given second-level folder.

| Channel | Description | Type | Mode |
|---|---|---|---|
| CD14 | CD14 staining, original single-channel image | original | gs |
| | ROF filtered cartoon derived from original | cartoon | gs |
| | mask highlighting the segmented macrophages | segment | bw |
| | mask highlighting the convex hulls of the segmented macrophages | convhull | bw |
| | mask highlighting the segmented macrophages bearing CD163 staining as well | multiple | bw |
| CD163 | CD163 staining, original single-channel image | original | gs |
| | ROF filtered cartoon derived from original | cartoon | gs |
| | mask highlighting the segmented macrophages | segment | bw |
| | mask highlighting the convex hulls of the segmented macrophages | convhull | bw |
| | mask highlighting the segmented macrophages bearing CD14 staining as well | multiple | bw |
| Pax5 | Pax5 staining, original single-channel image | original | gs |
| | ROF filtered cartoon derived from original | cartoon | gs |
| | mask highlighting the segmented B-cells | segment | bw |
| | mask highlighting the convex hulls of the segmented B-cells | convhull | bw |
| DAPI | DAPI staining, original single-channel image | original | gs |
| | ROF filtered cartoon derived from original | cartoon | gs |
| | mask representing the evaluation subregion | evalmask | bw |
| | mask highlighting the segmented cell nuclei | segment | bw |
| | mask highlighting the convex hulls of the segmented cell nuclei | convhull | bw |

## Dataset structure

Image data are organized by tissue specimens (top-level folders) and tiles (second-level folders), the latter ones ordered by position. Top-level folders are named `specimen_01`, … , `specimen_44`; second-level folders are named e.g. `specimen_01_tile_01_01`, … , `specimen_01_tile_09_08`. Within each second-level folder, 19 image files in greyscale ("gs") or black-and-white ("bw") mode are stored in losslessly compressed `.png` format. Table 1 and Figure 1 summarize the different images available at a given tile. The filenames are built as `specimen_xx_tile_yy_zz_channel_[CD14, CD163, Pax5, DAPI]_type_[original, cartoon, segment, convhull, multiple, evalmask]_mode_[gs, bw].png`. Size of losslessly compressed `.png` image files has been minimized by application of OptiPNG routine [8]. Moreover, a logfile named `specimen_xx_tile_yy_zz__logfile.txt` is provided, containing detailed information about procedures, parameters and results of automated segmentation.

## Reuse potential

Although there is a vast number of publications concerned with the composition of tumor microenvironment in various types of lymphoma disease, image datasets of IHC stained cancer tissue are rarely publicly accessible if at all, cf. the discussion in [9]. Most data generated for the purpose of such analyses are not findable or not even accessible. For example, the Genomic Data Commons Data Portal of the National Cancer Institute [10, 11] currently lists only 48 cases of mature B-cell lymphoma with an image of a HE-stained slide available, while IHC stainings are missing at all. In this situation, the image dataset presented in this note constitutes a document of interest in itself.

We will outline the most important options for further use of the data. First, it allows for a detailed morphometrical investigation of the imaged macrophages and B-cells with respect to distribution of geometrical parameters as size, diameter, perimeter, etc., as well as to overall shape patterns. Second, the data may be used for validation, calibration and comparison of cell segmentation methods (manual, automated) and related software packages, making available a large reference dataset together with the output of a reference method as described in [1]. In particular, the original images as well as the segmentations presented here could be used for the generation

of a sufficiently large training set for automated macrophage detection by machine learning methods. Third, the data may be used for study of co-localization and clustering of macrophages and B-cells within lymphoma tissue and cancer microenvironment, employing appropriate methods of point-pattern statistics [12, 13]. Note that the segmentation masks provided here are well-suited for a further processing of the obtained features (e.g. extraction of barycenters, replacement of the features by circles or squares of equal size). Finally, the dataset enables a closer study of the double-stained macrophage subpopulation.

To illustrate the described reuse potential, we include a set of composite figures, each combining information from several separate images. Figure 2.A shows an original image at CD14 channel (greyscale, contrast enhanced by factor 3.5, inverted) with superimposition of the mask of the evaluation subregion, as obtained from the DAPI channel (light blue), and the segmentation of the CD14-stained macrophages (olive green). Figure 2.B shows the same tile as imaged at the Pax5 channel (greyscale, inverted) with superimposition of the cell nuclei segmentation from DAPI channel (blue, convex hulls) and the segmentation of the CD163-stained macrophages (dark yellow). In Figure 2.C, for the same tile, both macrophage segmentations (olive green resp. dark yellow, convex hulls) are combined in order to reveal double-stained parts (light yellow). In Figure 2.D, we superimposed to Figure 2.C the segmentation of B-cells from the Pax5 channel (magenta resp. grey, convex hulls). Observe that some B-cells are positioned inside of macrophages, indicating that they are engulfed by the macrophages for phagocytosis. It is obvious that co-localization and clustering patterns as empirically noticeable here must be investigated on a sound base of statistical methodology.

## Availability of supporting data

All image data are publicly accessible at the Leipzig Health Atlas (LHA) repository [14] and can be reached from the address [15]. Each top-level folder can be downloaded as `.zip` file and bears a separate identifier, e.g. `https://health-atlas.de/lha/7WT490WEG2-7` within the repository, see Table 2. Two folders with total size larger than 1 GB (Nos. 04 and 44) have been splitted into a pair of files.

**Table 2.** Datasets available at the Leipzig Health Atlas.

| Name | Size (MB) | Identifier |
|---|---|---|
| specimen_01.zip | 167 | 7WT490WEG2-7 |
| specimen_02.zip | 146 | 7WT4A21HRJ-0 |
| specimen_03.zip | 125 | 7WT4AVVFEX-0 |
| specimen_04a.zip | 644 | 7WT5EP6YKD-6 |
| specimen_04b.zip | 731 | 7WT5J3GEK2-8 |
| specimen_05.zip | 674 | 7WT5P9AE2F-3 |
| specimen_06.zip | 173 | 7WT5QAKG27-4 |
| specimen_07.zip | 422 | 7WT5TVU42M-3 |
| specimen_08.zip | 409 | 7WT5W48QCX-6 |
| specimen_09.zip | 291 | 7WT5XWD0GT-7 |
| specimen_10.zip | 380 | 7WT614A02V-9 |
| specimen_11.zip | 729 | 7WT64Y97CY-7 |
| specimen_12.zip | 372 | 7WT67E4C8A-0 |
| specimen_13.zip | 154 | 7WT68MRXMT-1 |
| specimen_14.zip | 290 | 7WT69YN7KV-3 |
| specimen_15.zip | 128 | 7WT6C66UQ0-8 |
| specimen_16.zip | 150 | 7WT6GH4H0Q-7 |
| specimen_17.zip | 377 | 7WT6JKPKWV-5 |
| specimen_18.zip | 168 | 7WTH7R24UK-4 |
| specimen_19.zip | 109 | 7WTHAMJ6K4-2 |
| specimen_20.zip | 432 | 7WTJKQXW8X-6 |
| specimen_21.zip | 445 | 7WTJP21QET-7 |
| specimen_22.zip | 481 | 7WTJWJ3GY7-5 |
| specimen_23.zip | 343 | 7WTK8KRE71-9 |
| specimen_24.zip | 476 | 7WTKDD0PPG-7 |
| specimen_25.zip | 775 | 7WTKH27N4C-8 |
| specimen_26.zip | 658 | 7WTKKVQ8W1-5 |
| specimen_27.zip | 141 | 7WTKNFV28U-8 |
| specimen_28.zip | 232 | 7WTKPNXD2T-6 |
| specimen_29.zip | 567 | 7WTKT4NFNW-5 |
| specimen_30.zip | 345 | 7WTXJ4XWAD-0 |
| specimen_31.zip | 224 | 7WTXMW3UT9-1 |
| specimen_32.zip | 303 | 7WTXNVQ3X6-8 |
| specimen_33.zip | 341 | 7WTXQACWNG-8 |
| specimen_34.zip | 488 | 7WTXWFTC37-6 |
| specimen_35.zip | 294 | 7WTXYM32XF-3 |
| specimen_36.zip | 232 | 7WTY1FY9M5-0 |
| specimen_37.zip | 580 | 7WTY3EF3AA-2 |
| specimen_38.zip | 539 | 7WTY57UPU6-8 |
| specimen_39.zip | 907 | 7WTY81JGEU-0 |
| specimen_40.zip | 396 | 7WTY9TGTX1-3 |
| specimen_41.zip | 436 | 7WTYCTQU5A-8 |
| specimen_42.zip | 118 | 7WTYEECKGR-9 |
| specimen_43.zip | 619 | 7WTYGX9DNJ-2 |
| specimen_44a.zip | 524 | 7WTYPGF0CK-2 |
| specimen_44b.zip | 466 | 7WTYTQ9KUT-5 |

## Declarations

### List of abbreviations

CD14: a monocyte receptor protein; CD163: a macrophage receptor protein; DAPI: 4',6-diamidino-2-phenylindole; DLBCL: diffuse large B-cell lymphoma; HE: Haematoxylin-Eosin; IHC: immunohistochemical(ly); LHA: Leipzig Health Atlas; Pax5: a B-cell lineage specific activator protein; ROF: Rudin-Osher-Fatemi.

### Ethical Approval

Tissue usage is covered by a statement of the internal review board of the Medical Faculty of the Christian-Albrechts-University Kiel, Germany (No. 447/10).

### Consent for publication

Not applicable.

### Competing Interests

The authors declare that they have no competing interests.

### Funding

SR, RH and MW have been funded by BMBF project "MMML-Demonstrators", grants no. 031A428C and 031A428D.

### Author's Contributions

MW performed the image processing and wrote the manuscript. SR performed the IHF staining and image generation. RH curated the large-size image datasets and managed the storage within the Leipzig Health Atlas repository. WK identified the cohort. UDB contributed to the Context, Methods and Reuse potential sections. All authors read and approved the final manuscript.

## Acknowledgements

The authors would like to thank Dana Germer and Charlotte Botz-von Drathen for their excellent technical support.

## References

1. Wagner M, Hänsel R, Reinke S, Richter J, Altenbuchinger M, Braumann UD, Spang R, Löffler M, Klapper W. Automated macrophage counting in DLBCL tissue samples: a ROF filter based approach. Biol Proc Online 21 (2019) : 13 (electronically published)
2. Swerdlow SH, Campo E, Harris NL, Jaffe ES, Pileri SA, Stein H, Thiele J, Vardiman, JW (Eds). WHO Classification of Tumours of Haematopoietic and Lymphoid Tissues. WHO Classification of Tumours, Vol. 2. International Agency for Research on Cancer; Lyon 2017. 4th, rev. ed.
3. Scott DW, Gascoyne RD. The tumour microenvironment in B cell lymphomas. Nat Rev Cancer 14 (2014) : 517 – 534
4. Scott DW, Wright GW, Williams PM, Lih CJ, Walsh W, Jaffe ES, Rosenwald A, Campo E, Chan WC, Connors JM, Smeland EB, Mottok A, Braziel RM, Ott G, Delabie J, Tubbs RR, Cook JR, Weisenburger DD, Greiner TC, Glinsmann-Gibson BJ, Fu K, Staudt LM, Gascoyne RD, Rimsza LM. Determining cell-of-origin subtypes of diffuse large B-cell lymphoma using gene expression in formalin-fixed paraffin embedded tissue. Blood 123 (2014) : 1214 – 1217
5. Lozanski G, Pennell M, Shana'ah A, Zhao W, Gewirtz A, Racke F, Hsi E, Simpson S, Mosse C, Alam S, Swierczynski S, Hasserjian RP, Gurcan MN. Inter-reader variability in follicular lymphoma grading: conventional and digital reading. J Pathol Inform 4 (2013) : 30
6. Deroulers C, Ameisen D, Badoual M, Gerin C, Granier A, Lartaud M. Analyzing huge pathology images with open source software. Diagnostic Pathology 8 (2013) : 92
7. Narendra PM, Fitch RC. Real-time adaptive contrast enhancement. IEEE Trans Pattern Analysis Machine Int 3 (1981) : 655 – 661
8. `http://optipng.sourceforge.net` (accessed 03.09.2019)
9. Kostopoulos S, Ravazoula P, Asvestas P, Kalatzis I, Xenogiannopoulos G, Cavouras D, Glotsos D. Development of a reference image collection library for histopathology image processing, analysis and decision support systems research. J Digit Imaging 30 (2017) : 287 – 295
10. `https://portal.gdc.cancer.gov/repository` (accessed 03.09.2019)
11. Cooper LAD, Demicco EG, Saltz JH, Powell RT, Rao A, Lazar AJ. PanCancer insights from The Cancer Genome Atlas: the pathologist's perspective. J Pathol 244 (2018) : 512 – 524
12. Ripley BD. Spatial Statistics. Wiley; New York 1981
13. Møller J, Waagepetersen RP. Modern statistics for spatial point processes. Scand J Statistics 34 (2007) : 643 – 684
14. Meineke FA, Löbe M, Stäubert S. Introducing technical aspects of research data management in the Leipzig Health Atlas. Stud Health Technol Inform 247 (2018) : 426 – 430
15. `https://health-atlas.de/lha/7XWCUQPR8K-8` (accessed 03.09.2019)

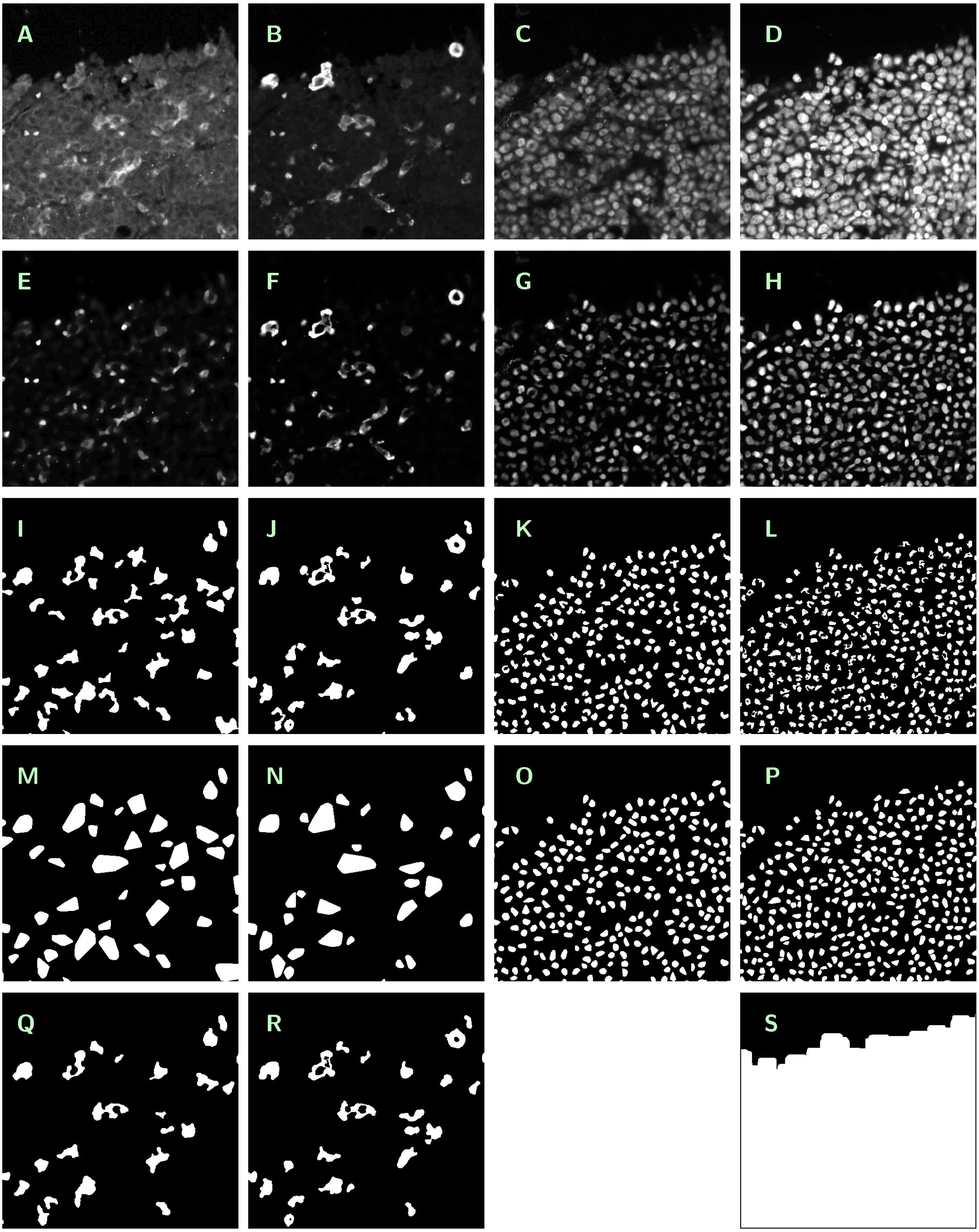


**Figure 1.** Summary of images available at a given tile (cutouts of 500 × 500 px size). Contrast enhanced in A by factor 3.5, in E by factor 7 and in F, G and H by factor 2.
*Originals (A–D).* A — `specimen_02_tile_01_06_channel_CD14_type_original_mode_gs.png`, B — `specimen_02_tile_01_06_channel_CD163_type_original_mode_gs.png`, C — `specimen_02_tile_01_06_channel_Pax5_type_original_mode_gs.png`, D — `specimen_02_tile_01_06_channel_DAPI_type_original_mode_gs.png`,
*Cartoons (E–H).* E — `specimen_02_tile_01_06_channel_CD14_type_cartoon_mode_gs.png`, F — `specimen_02_tile_01_06_channel_CD163_type_cartoon_mode_gs.png`, G — `specimen_02_tile_01_06_channel_Pax5_type_cartoon_mode_gs.png`, H — `specimen_02_tile_01_06_channel_DAPI_type_cartoon_mode_gs.png`,
*Segmentations (I–L).* I — `specimen_02_tile_01_06_channel_CD14_type_segment_mode_bw.png`, J — `specimen_02_tile_01_06_channel_CD163_type_segment_mode_bw.png`, K — `specimen_02_tile_01_06_channel_Pax5_type_segment_mode_bw.png`, L — `specimen_02_tile_01_06_channel_DAPI_type_segment_mode_bw.png`,
*Convex hulls (M–P).* M — `specimen_02_tile_01_06_channel_CD14_type_convhull_mode_bw.png`, N — `specimen_02_tile_01_06_channel_CD163_type_convhull_mode_bw.png`, O — `specimen_02_tile_01_06_channel_Pax5_type_convhull_mode_bw.png`, P — `specimen_02_tile_01_06_channel_DAPI_type_convhull_mode_bw.png`,
*Various (Q–S).* Q — `specimen_02_tile_01_06_channel_CD14_type_multiple_mode_bw.png`, R — `specimen_02_tile_01_06_channel_CD163_type_multiple_mode_bw.png`, S — `specimen_02_tile_01_06_channel_DAPI_type_evalmask_mode_bw.png`.

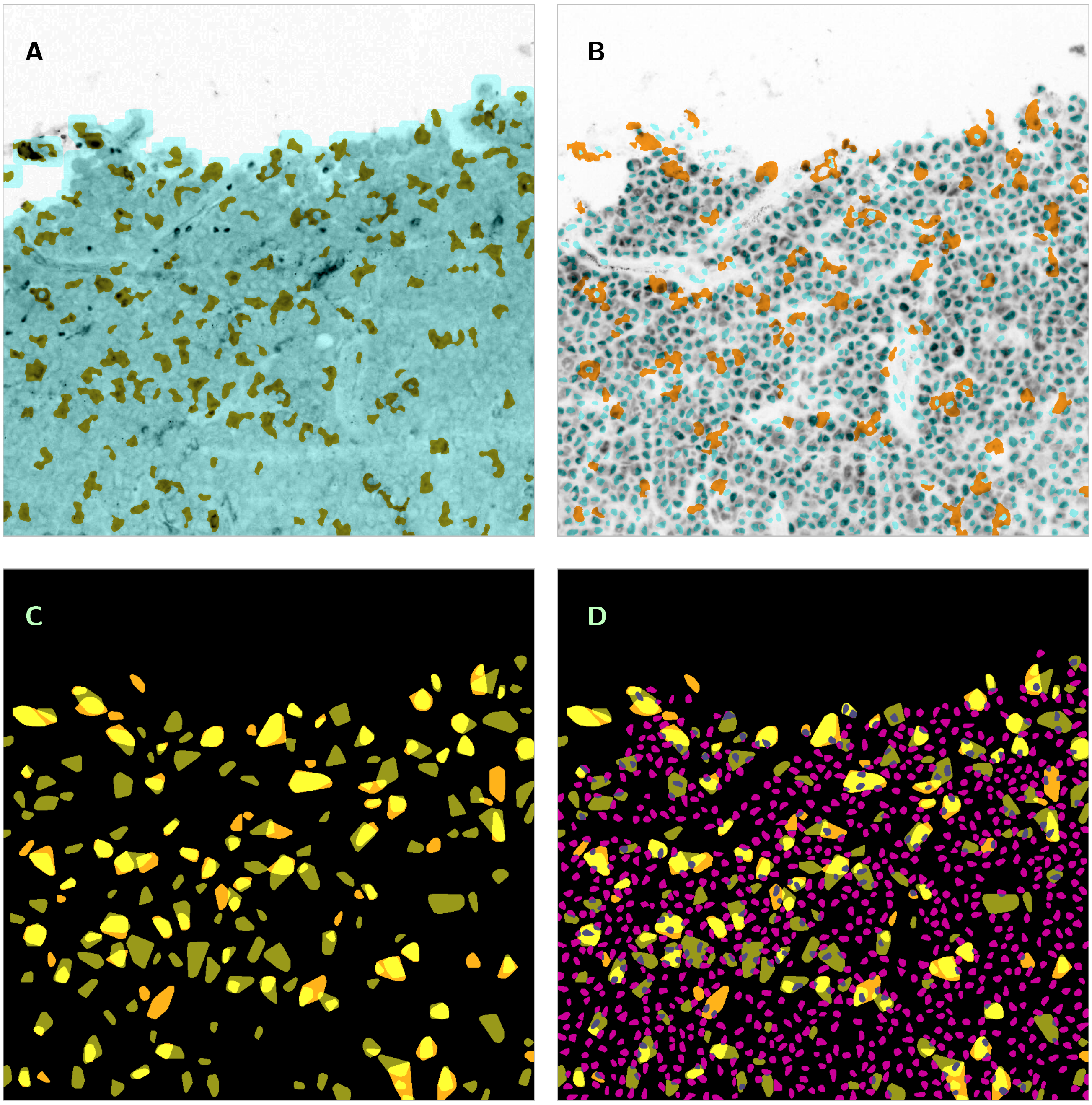


**Figure 2.** Examples of combined information from several images (see detailed description above). Image size is 1000 × 1000 px (450 × 450 µm$^2$). Composed images are based on `specimen_02_tile_01_06_` ...

A — `channel_CD14_type_original_mode_gs.png` / `channel_DAPI_type_evalmask_mode_bw.png` / `channel_CD14_type_segment_mode_bw.png`,

B — `channel_Pax5_type_original_mode_gs.png` / `channel_DAPI_type_convhull_mode_bw.png` / `channel_CD163_type_segment_mode_bw.png`,

C — `channel_CD14_type_convhull_mode_bw.png` / `channel_CD163_type_convhull_mode_bw.png`,

D — `channel_CD14_type_convhull_mode_bw.png` / `channel_CD163_type_convhull_mode_bw.png` / `channel_Pax5_type_convhull_mode_bw.png`.

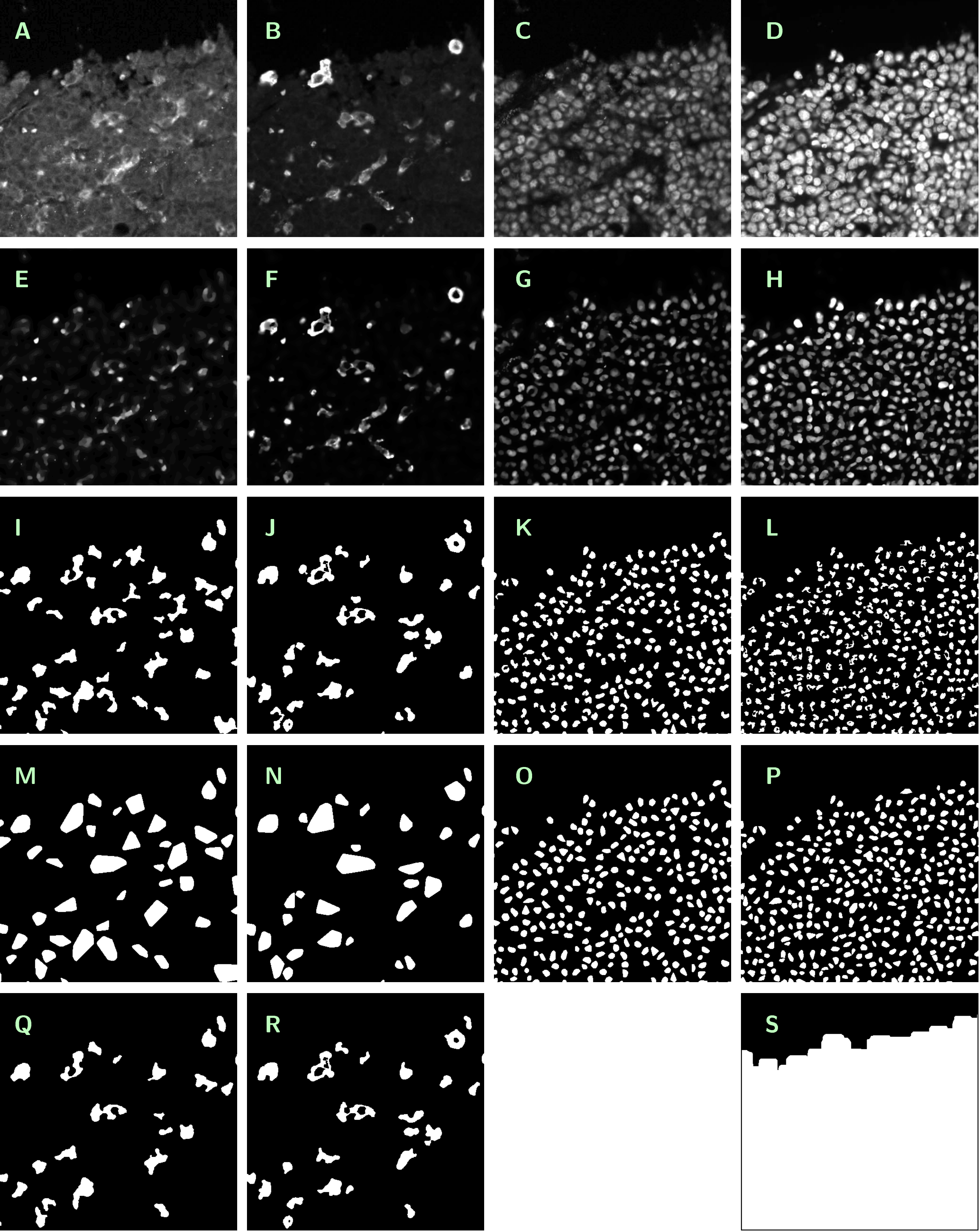
A
B
C
D
E
F
G
H
I
J
K
L
M
N
O
P
Q
R
S

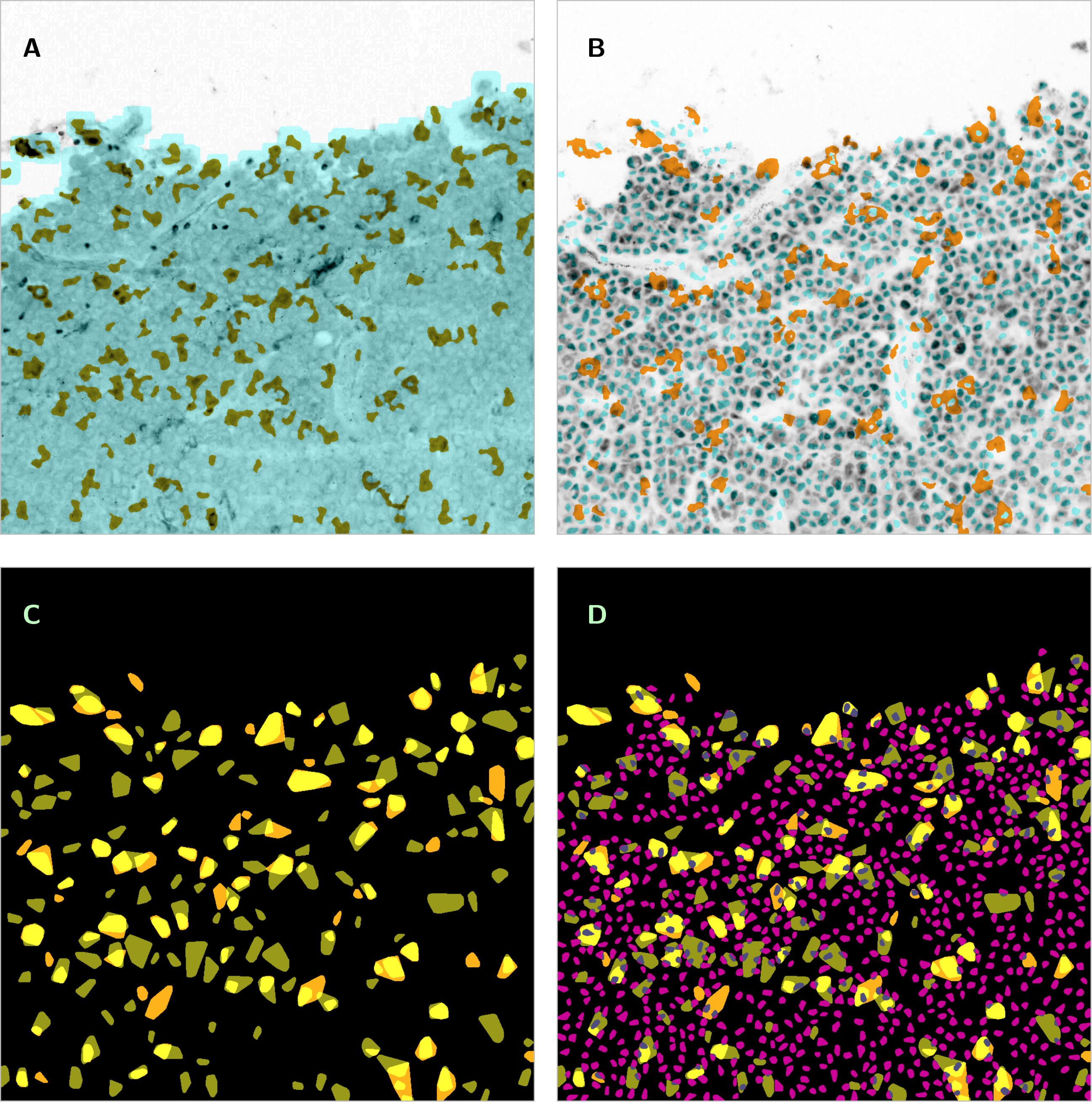
A
B
C
D

Cover letter Click here to download Personal Cover yh_cover.pdf

*Leipzig, September 04, 2019*

*Dr. rer. nat. habil. Marcus Wagner*
*Institute for Medical Informatics, Statistics and Epidemiology (IMISE)*
*University of Leipzig*
*Härtelstr. 16-18*
*D-04107 Leipzig, Germany*
*marcus.wagner@imise.uni-leipzig.de*

*GigaScience*
*Editor-in-Chief*
*Laurie Goodman, PhD*

*Dear Dr. Goodman,*

*hereby we submit our manuscript*

*"An image dataset related to automated macrophage detection in immunostained lymphoma tissue samples"*

*for publication as a Data Note in GigaScience. In our view, the note fits well into the aims and scope of the journal, making a well-curated image dataset with considerable reuse potential publicly findable, accessible und usable. Data presented here were generated as a part of an accompanying publication, which has recently appeared. They are hosted in the Leipzig Health Atlas repository and published there under CC0 license.*

*With best regards,*
*on behalf of the authors*

*Marcus Wagner.*
