# Supplementary material for: An image dataset related to automated macrophage detection in immunostained lymphoma tissue samples
Source: Gigascience. 2020 Mar 12;9(3):giaa016. doi: 10.1093/gigascience/giaa016 (PMC7066390; doi:10.1093/gigascience/giaa016)
Supplement: giaa016_GIGA-D-19-00311_Revision_2 [file giaa016_giga-d-19-00311_revision_2.pdf]

GigaScience

An image dataset related to automated macrophage detection in immunostained lymphoma tissue samples

--Manuscript Draft--

**Manuscript Number:**	GIGA-D-19-00311R2
**Full Title:**	An image dataset related to automated macrophage detection in immunostained lymphoma tissue samples
**Article Type:**	Data Note
**Funding Information:**	BMBF (031A428D) — Dr. Marcus Wagner, Mr. René Hänsel<br>BMBF (031A428C) — Dr. Sarah Reinke
**Abstract:**	Background: We present an image dataset related to automated segmentation and counting of macrophages in diffuse large B-cell lymphoma {(DLBCL)} tissue sections. For the classification of DLBCL subtypes as well as for as for providing a prognosis of the clinical outcome, the analysis of the tumor microenvironment and, particularly, of the different types and functions of tumor-associated macrophages, is indispensable. Until now, however, most information about macrophages is obtained either in a completely indirect way by gene expression profiling or by manual counts in immunohistochemically (IHC) fluorescence stained tissue samples while automated recognition of single IHC stained macrophages remains a difficult task. In an accompanying publication, a reliable approach to this problem has been established, and a large set of related images has been generated and analyzed.<br><br>Results: Provided image data comprise a) fluorescence microscopy images of 44 multiple immunohistostained DLBCL tumor subregions, captured at four channels corresponding to CD14, CD163, Pax5 and DAPI; b) "cartoon-like" TV-filtered versions of these images, generated by Rudin-Osher-Fatemi (ROF) denoising; c) an automatically generated mask of the evaluation subregion, based on information from the DAPI channel, and d) automatically generated segmentation masks for macrophages, B-cells and all cell nuclei, using information from CD14, CD163, Pax5 and DAPI channels, respectively.<br><br>Conclusions: A large set of IHC stained DLBCL specimens is provided together with segmentation masks for different cell populations generated by a reference method for automated image analysis, thus featuring considerable reuse potential.
**Corresponding Author:**	Marcus Wagner<br>Universitat Leipzig<br>Leipzig, GERMANY
**Corresponding Author Secondary Information:**	
**Corresponding Author's Institution:**	Universitat Leipzig
**Corresponding Author's Secondary Institution:**	
**First Author:**	Marcus Wagner
**First Author Secondary Information:**	
**Order of Authors:**	Marcus Wagner
	Sarah Reinke
	René Hänsel
	Wolfram Klapper
	Ulf-Dietrich Braumann

Powered by Editorial Manager® and ProduXion Manager® from Aries Systems Corporation

Order of Authors Secondary Information:	
Response to Reviewers:	The required changes and insertions have been made. Thank you for your assistance with RRIDs.<br>ORCIDs of all authors are included in a separate file as part of the submission.
Additional Information:	
Question	Response
Are you submitting this manuscript to a special series or article collection?	No
**Experimental design and statistics**<br><br>Full details of the experimental design and statistical methods used should be given in the Methods section, as detailed in our Minimum Standards Reporting Checklist. Information essential to interpreting the data presented should be made available in the figure legends.<br><br>Have you included all the information requested in your manuscript?	Yes
**Resources**<br><br>A description of all resources used, including antibodies, cell lines, animals and software tools, with enough information to allow them to be uniquely identified, should be included in the Methods section. Authors are strongly encouraged to cite Research Resource Identifiers (RRIDs) for antibodies, model organisms and tools, where possible.<br><br>Have you included the information requested as detailed in our Minimum Standards Reporting Checklist?	Yes
**Availability of data and materials**<br><br>All datasets and code on which the conclusions of the paper rely must be either included in your submission or deposited in publicly available repositories (where available and ethically appropriate), referencing such data using	Yes

a unique identifier in the references and in the “Availability of Data and Materials” section of your manuscript.<br><br>Have you have met the above requirement as detailed in our Minimum Standards Reporting Checklist?	

Powered by Editorial Manager® and ProduXion Manager® from Aries Systems Corporation

LaTeX - Main Document (TeX file)
Click here to access/download;LaTeX - Main Document (TeX file);yh_006.tex

Placeholder for OUP logo ./oup.pdf

Placeholder for journal logo ./gigascience-logo.pdf

GigaScience, 2019, 1–7

doi: xx.xxxx/xxxx
Manuscript in Preparation
Data Note

DATA NOTE

An image dataset related to automated macrophage detection in immunostained lymphoma tissue samples

Marcus Wagner[1,*,†], Sarah Reinke[2,†], René Hänsel[1], Wolfram Klapper[2] and Ulf-Dietrich Braumann[3]

[1]Institute for Medical Informatics, Statistics and Epidemiology (IMISE), University of Leipzig, Härtelstr. 16-18, D-04107 Leipzig, Germany and [2]Department of Pathology, Hematopathology Section and Lymph Node Registry, University of Kiel / University Hospital Schleswig-Holstein, Arnold-Heller-Str. 3, Haus 14, D-24105 Kiel, Germany and [3]Faculty of Engineering, Leipzig University of Applied Sciences (HTWK), P. O. B. 30 11 66, D-04251 Leipzig, Germany / Fraunhofer Institute for Cell Therapy and Immunology (IZI), Perlickstr. 1, D-04103 Leipzig, Germany

[*] Correspondence address. Institute for Medical Informatics, Statistics and Epidemiology (IMISE), University of Leipzig, Härtelstr. 16-18, D-04107 Leipzig, Germany. E-mail: marcus.wagner@imise.uni-leipzig.de
[†] Contributed equally.

Abstract

Background: We present an image dataset related to automated segmentation and counting of macrophages in diffuse large B-cell lymphoma (DLBCL) tissue sections. For the classification of DLBCL subtypes as well as for as for providing a prognosis of the clinical outcome, the analysis of the tumor microenvironment and, particularly, of the different types and functions of tumor-associated macrophages, is indispensable. Until now, however, most information about macrophages is obtained either in a completely indirect way by gene expression profiling or by manual counts in immunohistochemically (IHC) fluorescence stained tissue samples while automated recognition of single IHC stained macrophages remains a difficult task. In an accompanying publication, a reliable approach to this problem has been established, and a large set of related images has been generated and analyzed.
Results: Provided image data comprise a) fluorescence microscopy images of 44 multiple immunohistostained DLBCL tumor subregions, captured at four channels corresponding to CD14, CD163, Pax5 and DAPI; b) "cartoon-like" TV-filtered versions of these images, generated by Rudin-Osher-Fatemi (ROF) denoising; c) an automatically generated mask of the evaluation subregion, based on information from the DAPI channel, and d) automatically generated segmentation masks for macrophages, B-cells and all cell nuclei, using information from CD14, CD163, Pax5 and DAPI channels, respectively.
Conclusions: A large set of IHC stained DLBCL specimens is provided together with segmentation masks for different cell populations generated by a reference method for automated image analysis, thus featuring considerable reuse potential.

Key words: lymphoma, DLBCL, macrophage, multiple immunohistochemical staining, automated cell counting, ROF filtering, floating threshold, rule-based detection, image dataset

Compiled on: February 7, 2020.
Draft manuscript prepared by the author.

Data Description

Context

We present an image dataset generated as a part of an accompanying publication, which is concerned with method development and comparison for automated segmentation and counting of macrophages in diffuse large B-cell lymphoma (DLBCL) tissue sections [1]. DLBCL is an aggressive cancer disease which is characterized by a large heterogeneity of pathological, clinical and biological features [2]. Therefore, a crucial step for the classification of DLBCL subtypes as well as for providing a prognosis of the clinical outcome is the analysis of the tumor microenvironment in terms of counts, local distributions and functions of the different cell populations and, particularly, of the tumor-associated macrophages occuring there [3].

Until now, most information about macrophages is obtained either by gene expression profiling [4] or by manual counts in immunohistochemically (IHC) stained tissue microarrays or high-power fields, thus either gathering information in a completely indirect way or accepting extreme subsampling rates [5]. A reliable approach for fully automated segmentation, identification and counting of IHC stained macrophages within whole tissue slides has been addressed in [1].

Our dataset contains monochrome fluorescence microscopy images of 44 DLBCL tissue samples wherein different macrophage populations (using antibodies against CD14 and CD163) and B-cells (using antibody against Pax5) as well as all cell nuclei (using DAPI) have been stained and imaged at different wavelengths. Further, we supply processed images, comprising "cartoon-like" TV-filtered images (generated by Rudin-Osher-Fatemi filtering) as well as results of the automated macrophage segmentation. For this publication, we completed these data by automated segmentation of B-cells and the cell nuclei.

Methods

a) Preparation and staining of DLBCL tissue. From the files of the Lymph Node Registry Kiel, 44 DLBCL biopsy specimens have been selected. For every specimen, from formalin-fixed paraffin-embedded tissue a slice of 2 μm thickness has been obtained. In order to detect specific macrophages and its relation to B-cells, a triple IHC staining has been done, using primary antibodies against CD14 (Cell Marque, Cat# 114R-14, RRID: AB_2827391; 1:10), CD163 (Novus, Cat# NB110-59935, RRID: AB_892323; 1:100) and Pax5 (Santa Cruz Biotechnology, Cat# sc-1974, RRID: AB_2159678; 1:100) labelled with donkey anti rabbit Alexa 488, donkey anti mouse Alexa 555 and donkey anti goat Alexa 647 (all from Invitrogen, Thermo Fisher Scientific, Waltham, MA, USA; 1:100) as secondary antibodies. Subsequently, the slices have been incubated with DAPI (Invitrogen, Thermo Fisher Scientific, Waltham, MA, USA; 1:5000) and cover-slipped with mounting medium. Use of tissue was in accordance with the guidelines of the internal review board of the Medical Faculty of the Christian-Albrechts-University Kiel, Germany (No. 447/10).

b) Selection of tumor subregions and image acquisition. Within every tissue sample, the tumor area was defined and marked by a pathologist based on inspection of conventional Haematoxylin-Eosin (HE) staining in a neighboring reference slice. Subsequently, within the IHC stained slice, a rectangular subregion of the tumor area has been selected, taking care for acceptable tissue and staining quality. Maximum size of tumor subregions is 10 mm^2.

Images of tumor subregions within the IHC stained slides have been captured by Hamamatsu Nanozoomer 2.0 RS slide scanner (Hamamatsu Photonics, Ammersee, Germany) with 20 × magnification at four wavelengths, resulting in single images for the CD14, CD163, Pax5 and DAPI channels, respectively, which were saved in `.ndpi` output format with default settings as used in clinical trial routine. Note that, at this point, moderate built-in compression by imaging device was accepted. Single-channel raw images have been converted into `.tif` format without further compression and sliced into tiles of 1000 × 1000 px format (at right and lower border, the sizes may be smaller), using the software package ImageJ with the extension ndpitools [6]. The resulting monochrome images have been further converted from RGB into greyscale mode using the modulus of the RGB vector and finally saved in losslessly compressed `.png` format. We refer to them as to images of type "original". Let us remark that image acquisition and tiling have been performed in such a way that no spatial misalignment between the scans at the different wavelengths occurred. Pixel size is $0.45 \times 0.45\ \mu\mathrm{m}^2$ in all images.

c) Image processing. For every tile, the segmentation method from [1] has been applied to the CD14, CD163, Pax5 and DAPI channel images, resulting in ROF-filtered images (saved as type "cartoon"), a mask for the evaluation subregion within the tile, indicating the presence of tissue at all, as inferred from DAPI channel information (saved as type "evalmask"), and segmentations of macrophages within the CD14 and CD163 channels (saved as type "segment"). Due to the large inhomogeneity of IHC staining, even across a single target macrophage, we provide two further masks containing the convex hulls of the segmented features instead of the features themselves (saved as type "convhull"). The segmentation masks for double-stained macrophages are saved as type "multiple". For a general description of the ROF filter based segmentation method, we refer to [1]. Here, we describe in more detail the generation of segmentations for the Pax5 and DAPI channels, which are new in this paper.

Let us recall the notation from [1] where the indices i and j count the current intensity threshold and the features to be inspected at this stage, $s(F_j)$, $c(F_j)$ and $r(F_j)$ denote the size of a feature F_j itself, the size of its convex hull and the ratio of the principal axes' lengths of the smallest ellipse covering the feature, respectively. s_{min}, s_{max}, c_{max} and r_{max} denote the minimal and maximal feature size (in px), the maximal area excess of the convex hull (in percent) and the maximal ratio of axes, respectively.

In order to obtain a segmentation of the DAPI channel, the ROF-filtered image has been further subjected to a local Narendra-Fitch contrast enhancement [7]

$$p(k,l)_{enhanced} = m(k,l) + \frac{c}{\sigma(k,l)} \cdot \left(p(k,l)_{original} - m(k,l) \right) \quad (1)$$

where $c > 0$ is a weight parameter and $m(k,l)$, $\sigma(k,l)$ denote the mean and standard deviation of the intensities within a subregion centered at the pixel $p(k,l)_{original}$, respectively. We used $c = 0.75$ and a square subregion of 11×11 px size. Then, in a first run, Steps 3 – 10 of the ROF filter based segmentation have been applied, using the bounds $s_{min} = 60$ and $s_{max} = 119$ for the feature size but modifying geometrical rule No. 3) for feature classification from [1] as follows: If $s_{min} \leq s(F) \leq s_{max}$ then test whether the feature satisfies both of the criteria 3b) $r(F_j) \leq r_{max}$ (the feature is not too elongated) and 3d) $c(F_j)/s(F_j) \leq 1 + c_{max}/100$ (the deviation from circular shape is bounded from above). If yes, save the feature F_j into the output mask, interpreting it as a cell nucleus, and mask it in $I^{(3)}(i)$. If not then neglect the feature and mask it in $I^{(3)}(i)$ as well. Here, we used the parameter values $r_{max} = 2.5$ and $c_{max} = 150$. In a second run, Steps 3 – 10 of the ROF filter based segmentation have been repeated with the parameter settings $s_{min} = 120$ and

s_{max} = 180, using again the described modification of rule No. 3) but saving only those features into the output mask which are completely disjoint to the output of the first run. Finally, the results of both runs have been combined into a single mask (saved as type "segment"). Within a further result mask of type "convhull", the convex hulls of the detected features have been stored.

For the segmentation of the B-cells, the ROF-filtered image of the Pax5 channel has been subjected to a moderate Narendra-Fitch contrast enhancement as well, using the parameter c = 0.1 and a square subregion of 15×15 px size. To the result, Steps 3 – 10 of the ROF filter based segmentation have been applied, using the bounds s_{min} = 80 and s_{max} = 159 as well as the described modification of rule No. 3) with parameters r_{max} = 2.5 and c_{max} = 150 but saving into the output mask (of type "segment") only features which intersection with the convex hull of some cell nucleus, as obtained in the segmentation of the DAPI channel, is nonempty. Thus, numerous artifacts appearing in the Pax5 staining will be excluded. Again, the convex hulls of the dectected features have been stored within a further mask of type "convhull".

Dataset structure

Image data are organized by tissue specimens (top-level folders) and tiles (second-level folders), the latter ones ordered by position. Top-level folders are named `specimen_01`, ... , `specimen_44`; second-level folders are named e.g. `specimen_01_tile_01_01`, ... , `specimen_01_tile_09_08`. Within each second-level folder, 19 image files in greyscale ("gs") or black-and-white ("bw") mode are stored in losslessly compressed `.png` format with 8-bit or 1-bit depth, respectively. Table 1 and Figure 1 summarize the different images available at a given tile. The filenames are built as `specimen_xx_tile_yy_zz_channel_[CD14, CD163, Pax5, DAPI]_type_[original, cartoon, segment, convhull, multiple, evalmask]_mode_[gs, bw].png`. Size of losslessly compressed `.png` image files has been minimized by application of OptiPNG routine [8]. Moreover, a logfile named `specimen_xx_tile_yy_zz__logfile.txt` is provided, containing detailed information about procedures, parameters and results of automated segmentation.

Table 1. Image files available within a given second-level folder.

Channel	Description	Type	Mode
CD14	CD14 staining, original single-channel image	original	gs
	ROF filtered image derived from original	cartoon	gs
	mask highlighting the segmented macrophages	segment	bw
	mask highlighting the convex hulls of the segmented macrophages	convhull	bw
	mask highlighting the segmented macrophages bearing CD163 staining as well	multiple	bw
CD163	CD163 staining, original single-channel image	original	gs
	ROF filtered image derived from original	cartoon	gs
	mask highlighting the segmented macrophages	segment	bw
	mask highlighting the convex hulls of the segmented macrophages	convhull	bw
	mask highlighting the segmented macrophages bearing CD14 staining as well	multiple	bw
Pax5	Pax5 staining, original single-channel image	original	gs
	ROF filtered image derived from original	cartoon	gs
	mask highlighting the segmented B-cells	segment	bw
	mask highlighting the convex hulls of the segmented B-cells	convhull	bw
DAPI	DAPI staining, original single-channel image	original	gs
	ROF filtered image derived from original	cartoon	gs
	mask representing the evaluation subregion	evalmask	bw
	mask highlighting the segmented cell nuclei	segment	bw
	mask highlighting the convex hulls of the segmented cell nuclei	convhull	bw

d) BLC2 scoring. For all specimens, a BCL2 score is available, see Table 2. It is based on BCL2-staining for tissue slides obtained from the same biopsy specimens as before but not necessarily adjacent to the slides used for the generation of the image data presented here. Staining was microscopically examined and semi-quantitatively scored by an experienced pathologist. Each stained slide was evaluated for the percentage of stained tumor cells by visual estimation in a representative tumor area. The estimated value was graded into following scores: 0 — all cells negative, 1 — up to 25% positive cells, 2 — 25% – 50% positive cells, 3 — 50% – 75% positive cells, 4 — over 75% positive cells.

Reuse potential

Although there is a vast number of publications concerned with the composition of tumor microenvironment in various types of lymphoma disease, image datasets of IHC stained cancer tissue are rarely publicly accessible if at all, cf. the discussion in [9]. Most data generated for the purpose of such analyses are not findable or not even accessible. For example, the Genomic Data Commons Data Portal of the National Cancer Institute [10, 11] currently lists only 48 cases of mature B-cell lymphoma with an image of a HE-stained slide available, while IHC stainings are missing at all. In this situation, the image

dataset presented in this note constitutes a document of interest in itself.

We will outline the most important options for further use of the data. First, it allows for a detailed morphometrical investigation of the imaged macrophages and B-cells with respect to the distribution of geometrical parameters as size, diameter, perimeter, etc., as well as to overall shape patterns. Second, the data may be used for validation, calibration and comparison of cell segmentation methods (manual, automated) and related software packages, making available a large reference dataset together with the output of a reference method as described in [1]. Note that, for these purposes, it is particularly adequate to use data admitting a routine quality level. Third, the original images as well as the segmentations presented here could be used for the generation of a sufficiently large training set for automated macrophage detection by machine learning methods. Fourth, the data may be used for study of co-localization and clustering of macrophages and B-cells within lymphoma tissue and cancer microenvironment, employing appropriate methods of point-pattern statistics [12, 13]. Finally, the dataset enables a closer study of the double-stained macrophage subpopulation. In order to facilitate a possible further processing of the obtained features (e.g. extraction of barycenters, replacement of the features by equally sized circles or squares), not only the masks for the segmented features themselves but as well for its convex hulls are provided.

To illustrate the described reuse potential, we include a set of composite figures, each combining information from several separate images. Figure 2.A shows an original image at CD14 channel (greyscale, original contrast-enhanced by factor 3.5 and inverted) with superimposition of the mask of the evaluation subregion, as obtained from the DAPI channel (light blue), and the segmentation of the CD14-stained macrophages (olive green). Figure 2.B shows the same tile as imaged at the Pax5 channel (greyscale, original inverted) with superimposition of the cell nuclei segmentation from DAPI channel (light blue, convex hulls) and the segmentation of the CD163-stained macrophages (dark yellow). In Figure 2.C, for the same tile, both macrophage segmentations (olive green or dark yellow, convex hulls) are combined in order to reveal double-stained parts (light yellow). In Figure 2.D, we superimposed to Figure 2.C the segmentation of B-cells from the Pax5 channel (magenta and grey, convex hulls). Observe that in Figs. 2.B and 2.D, some B-cells are positioned inside of macrophages, indicating that they are engulfed by the macrophages for phagocytosis (examples marked by arrows). It is obvious that co-localization and clustering patterns as empirically noticeable here must be investigated on a sound base of statistical methodology.

To improve reusability, BLC2 scores for the biopsy specimens are provided.

Table 2. BLC2 scores.

Score	Specimen No.
0	03, 05, 18, 32, 37, 43, 44
1	—
2	36
3	28
4	01, 02, 04, 06 – 17, 19 – 27, 29 – 31, 33 – 35, 38 – 42

Availability of supporting data

All image data are made publicly accessible under CC0 1.0 license at the Leipzig Health Atlas (LHA) repository [14] and can be reached from the address [15]. Each top-level folder can be downloaded as `.zip` file and bears a separate identifier, e.g. `https://health-atlas.de/lha/7YXMMFNPDG-0` within the repository, see Table 3. Two folders with total size larger than 1 GB (Nos. 04 and 44) have been splitted into a pair of files. Snapshots of the datasets are available in the GigaScience GigaDB repository as well [16].

Table 3. Datasets available at the Leipzig Health Atlas.

Name	Size (MB)	Identifier
specimen_01.zip	161	7YXMMFNPDG-0
specimen_02.zip	142	7YXXYUTPYN-9
specimen_03.zip	121	7YXY2MUWDK-3
specimen_04a.zip	630	7YXYECRXQM-0
specimen_04b.zip	709	7YXYY60JX7-9
specimen_05.zip	653	7YY08G00A0-4
specimen_06.zip	168	7YY0NHJXF8-2
specimen_07.zip	409	7YY0X073KU-7
specimen_08.zip	396	7YY146X8HE-4
specimen_09.zip	283	7YY19AWR7C-8
specimen_10.zip	368	8004FF6QR6-5
specimen_11.zip	708	8004RQHWHX-6
specimen_12.zip	360	800516PXMC-9
specimen_13.zip	150	8005NDPDNX-6
specimen_14.zip	283	8005QXY7QG-0
specimen_15.zip	124	8005TY4388-4
specimen_16.zip	146	8005X2U355-5
specimen_17.zip	364	80062H7C7J-4
specimen_18.zip	164	800HNVWTJX-5
specimen_19.zip	105	800HR9GPJE-5
specimen_20.zip	418	800J5AN4V1-7
specimen_21.zip	431	800JDAJXHV-6
specimen_22.zip	465	802X2RAVTV-8
specimen_23.zip	333	802X8VYQ27-0
specimen_24.zip	462	802XR67DWU-2
specimen_25.zip	749	802Y1JFKPQ-6
specimen_26.zip	635	803AHC5EAH-5
specimen_27.zip	137	803AHW6TD9-4
specimen_28.zip	225	803AU2NYKJ-8
specimen_29.zip	549	803C11PMP7-3
specimen_30.zip	334	803C4Q94NP-5
specimen_31.zip	217	803C809ERJ-6
specimen_32.zip	293	803CFU4J96-9
specimen_33.zip	330	803CJR62YA-8
specimen_34.zip	474	803NYKM0PY-9
specimen_35.zip	286	803PH07HQT-2
specimen_36.zip	225	803PJKT2JG-7
specimen_37.zip	563	803PKWG9XG-9
specimen_38.zip	524	803PPV4R44-8
specimen_39.zip	879	8044GTGCPG-1
specimen_40.zip	382	8044J0U5JC-0
specimen_41.zip	421	804GHX9A2E-8
specimen_42.zip	114	804GJF4HQ4-8
specimen_43.zip	596	804GY21PMN-9
specimen_44a.zip	506	804H6EM8W2-5
specimen_44b.zip	452	804H7C4T1P-0

Declarations

List of abbreviations

DAPI: 4',6-diamidino-2-phenylindole; DLBCL: diffuse large B-cell lymphoma; HE: Haematoxylin-Eosin; IHC: immunohistochemical(ly); LHA: Leipzig Health Atlas; ROF: Rudin-Osher-Fatemi; TV: total variation.

Ethical Approval

Tissue usage is covered by statement No. 447/10 of the internal review board of the Medical Faculty of the Christian-Albrechts-University Kiel, Germany.

Consent for publication

Not applicable.

Competing Interests

The authors declare that they have no competing interests.

Funding

SR, RH and MW have been funded by BMBF project "MMML-Demonstrators", grants no. 031A428C and 031A428D.

Author's Contributions

MW performed the image processing and wrote the manuscript. SR performed the IHF staining and image generation. RH curated the large-size image datasets and managed the storage within the Leipzig Health Atlas repository. WK identified the cohort. UDB contributed to the Context, Methods and Reuse potential sections. All authors read and approved the final manuscript.

Acknowledgements

The authors would like to thank Dana Germer and Charlotte Botz-von Drathen for their excellent technical support.

References

1. Wagner M, Hänsel R, Reinke S, Richter J, Altenbuchinger M, Braumann UD, Spang R, Löffler M, Klapper W. Automated macrophage counting in DLBCL tissue samples: a ROF filter based approach. Biol Proc Online 21 (2019) : 13 (electronically published)
2. Swerdlow SH, Campo E, Harris NL, Jaffe ES, Pileri SA, Stein H, Thiele J, Vardiman, JW (Eds). WHO Classification of Tumours of Haematopoietic and Lymphoid Tissues. WHO Classification of Tumours, Vol. 2. International Agency for Research on Cancer; Lyon 2017. 4th, rev. ed.
3. Scott DW, Gascoyne RD. The tumour microenvironment in B cell lymphomas. Nat Rev Cancer 14 (2014) : 517 – 534
4. Scott DW, Wright GW, Williams PM, Lih CJ, Walsh W, Jaffe ES, Rosenwald A, Campo E, Chan WC, Connors JM, Smeland EB, Mottok A, Braziel RM, Ott G, Delabie J, Tubbs RR, Cook JR, Weisenburger DD, Greiner TC, Glinsmann-Gibson BJ, Fu K, Staudt LM, Gascoyne RD, Rimsza LM. Determining cell-of-origin subtypes of diffuse large B-cell lymphoma using gene expression in formalin-fixed paraffin embedded tissue. Blood 123 (2014) : 1214 – 1217
5. Lozanski G, Pennell M, Shana'ah A, Zhao W, Gewirtz A, Racke F, Hsi E, Simpson S, Mosse C, Alam S, Swierczynski S, Hasserjian RP, Gurcan MN. Inter-reader variability in follicular lymphoma grading: conventional and digital reading. J Pathol Inform 4 (2013) : 30
6. Deroulers C, Ameisen D, Badoual M, Gerin C, Granier A, Lartaud M. Analyzing huge pathology images with open source software. Diagnostic Pathology 8 (2013) : 92
7. Narendra PM, Fitch RC. Real-time adaptive contrast enhancement. IEEE Trans Pattern Analysis Machine Int 3 (1981) : 655 – 661
8. `http://optipng.sourceforge.net` (accessed 06.02.2020)
9. Kostopoulos S, Ravazoula P, Asvestas P, Kalatzis I, Xenogiannopoulos G, Cavouras D, Glotsos D. Development of a reference image collection library for histopathology image processing, analysis and decision support systems research. J Digit Imaging 30 (2017) : 287 – 295
10. `https://portal.gdc.cancer.gov/repository` (accessed 06.02.2020)
11. Cooper LAD, Demicco EG, Saltz JH, Powell RT, Rao A, Lazar AJ. PanCancer insights from The Cancer Genome Atlas: the pathologist's perspective. J Pathol 244 (2018) : 512 – 524
12. Ripley BD. Spatial Statistics. Wiley; New York 1981
13. Møller J, Waagepetersen RP. Modern statistics for spatial point processes. Scand J Statistics 34 (2007) : 643 – 684
14. Meineke FA, Löbe M, Stäubert S. Introducing technical aspects of research data management in the Leipzig Health Atlas. Stud Health Technol Inform 247 (2018) : 426 – 430
15. `https://health-atlas.de/lha/7XWCUQPR8K-8` (accessed 06.02.2020)
16. Wagner M, Reinke S, Hänsel R, Klapper W, Braumann UD. Supporting data for "An image dataset related to automated macrophage detection in immunostained lymphoma tissue samples". GigaScience Database (2020), `http://dx.doi.org/10.5524/100703` (accessed 06.02.2020)

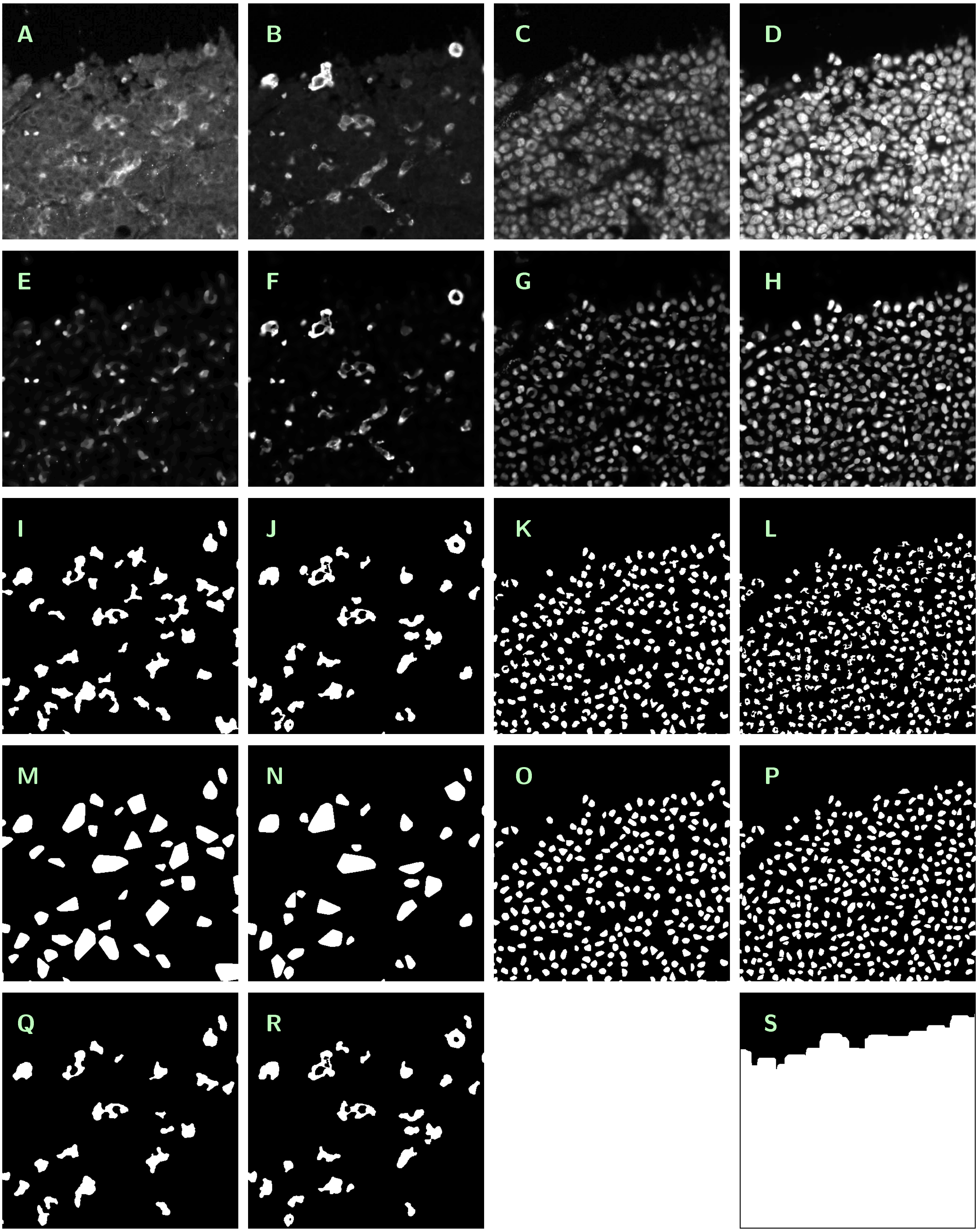

Figure 1. Summary of images available at a given tile (cutouts of 500 $\times$ 500 px size). Contrast enhanced in A by factor 3.5, in E by factor 7 and in F, G and H by factor 2.
Originals (A–D). A — `specimen_02_tile_01_06_channel_CD14_type_original_mode_gs.png`, B — `specimen_02_tile_01_06_channel_CD163_type_original_mode_gs.png`, C — `specimen_02_tile_01_06_channel_Pax5_type_original_mode_gs.png`, D — `specimen_02_tile_01_06_channel_DAPI_type_original_mode_gs.png`,
Cartoons (E–H). E — `specimen_02_tile_01_06_channel_CD14_type_cartoon_mode_gs.png`, F — `specimen_02_tile_01_06_channel_CD163_type_cartoon_mode_gs.png`, G — `specimen_02_tile_01_06_channel_Pax5_type_cartoon_mode_gs.png`, H — `specimen_02_tile_01_06_channel_DAPI_type_cartoon_mode_gs.png`,
Segmentations (I–L). I — `specimen_02_tile_01_06_channel_CD14_type_segment_mode_bw.png`, J — `specimen_02_tile_01_06_channel_CD163_type_segment_mode_bw.png`, K — `specimen_02_tile_01_06_channel_Pax5_type_segment_mode_bw.png`, L — `specimen_02_tile_01_06_channel_DAPI_type_segment_mode_bw.png`,
Convex hulls (M–P). M — `specimen_02_tile_01_06_channel_CD14_type_convhull_mode_bw.png`, N — `specimen_02_tile_01_06_channel_CD163_type_convhull_mode_bw.png`, O — `specimen_02_tile_01_06_channel_Pax5_type_convhull_mode_bw.png`, P — `specimen_02_tile_01_06_channel_DAPI_type_convhull_mode_bw.png`,
Various (Q–S). Q — `specimen_02_tile_01_06_channel_CD14_type_multiple_mode_bw.png`, R — `specimen_02_tile_01_06_channel_CD163_type_multiple_mode_bw.png`, S — `specimen_02_tile_01_06_channel_DAPI_type_evalmask_mode_bw.png`.

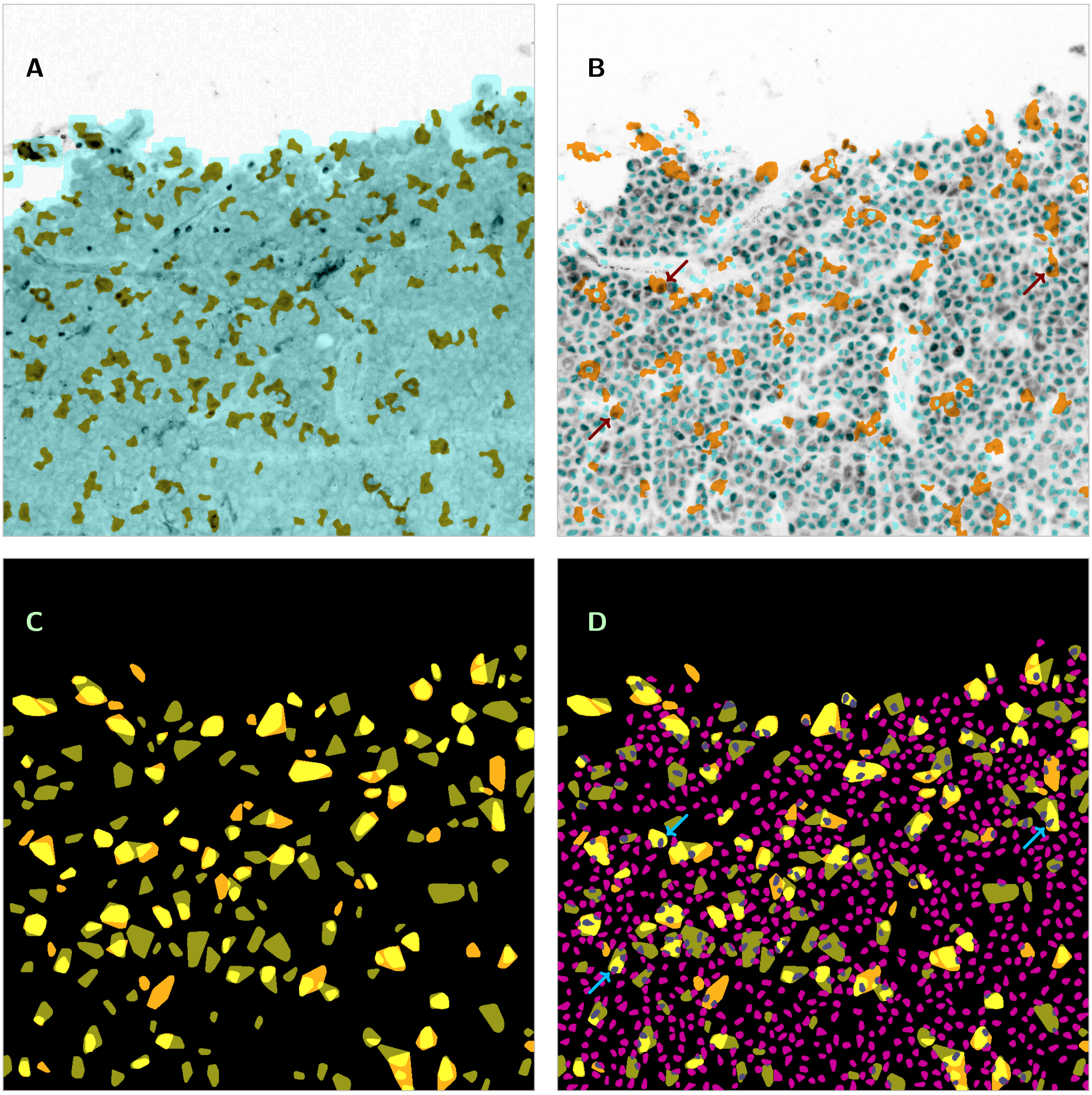

Figure 2. Examples of combined information from several images, based on `specimen_02_tile_01_06_...` Image size is 1000 × 1000 px (450 × 450 μm^2).
A — Original image at CD14 channel (greyscale, contrast enhanced by factor 3.5, inverted) (`channel_CD14_type_original_mode_gs.png`) with superimposition of the mask of the evaluation subregion, as obtained from the DAPI channel (light blue) (`channel_DAPI_type_evalmask_mode_bw.png`) and the segmentation of the CD14-stained macrophages (olive green) (`channel_CD14_type_segment_mode_bw.png`).
B — The same tile as imaged at the Pax5 channel (greyscale, inverted) (`channel_Pax5_type_original_mode_gs.png`) with superimposition of the cell nuclei segmentation from DAPI channel (light blue, convex hulls) (`channel_DAPI_type_convhull_mode_bw.png`) and the segmentation of the CD163-stained macrophages (dark yellow) (`channel_CD163_type_segment_mode_bw.png`). Examples of B-cells positioned inside of macrophages indicated by arrows.
C — Combination of both macrophage segmentations (olive green or dark yellow, convex hulls) for the same tile in order to reveal double-stained parts (light yellow) (`channel_CD14_type_convhull_mode_bw.png` / `channel_CD163_type_convhull_mode_bw.png`).
D — Segmentation of B-cells from the Pax5 channel (magenta and grey, convex hulls) (`channel_Pax5_type_convhull_mode_bw.png`) superimposed to Figure 2.C. Examples of B-cells positioned inside of macrophages indicated by arrows (the same cells as in Figure 2.B).

Manuscript Click here to access/download;Manuscript;yh_006.pdf

OXFORD (GIGA)^n SCIENCE

GigaScience, 2019, 1–7

doi: xx.xxxx/xxxx
Manuscript in Preparation
Data Note

DATA NOTE

An image dataset related to automated macrophage detection in immunostained lymphoma tissue samples

Marcus Wagner[1,*,†], Sarah Reinke[2,†], René Hänsel[1], Wolfram Klapper[2] and Ulf-Dietrich Braumann[3]

[1]Institute for Medical Informatics, Statistics and Epidemiology (IMISE), University of Leipzig, Härtelstr. 16-18, D-04107 Leipzig, Germany and [2]Department of Pathology, Hematopathology Section and Lymph Node Registry, University of Kiel / University Hospital Schleswig-Holstein, Arnold-Heller-Str. 3, Haus 14, D-24105 Kiel, Germany and [3]Faculty of Engineering, Leipzig University of Applied Sciences (HTWK), P. O. B. 30 11 66, D-04251 Leipzig, Germany / Fraunhofer Institute for Cell Therapy and Immunology (IZI), Perlickstr. 1, D-04103 Leipzig, Germany

[*] Correspondence address. Institute for Medical Informatics, Statistics and Epidemiology (IMISE), University of Leipzig, Härtelstr. 16-18, D-04107 Leipzig, Germany. E-mail: marcus.wagner@imise.uni-leipzig.de
[†] Contributed equally.

Abstract

Background: We present an image dataset related to automated segmentation and counting of macrophages in diffuse large B-cell lymphoma (DLBCL) tissue sections. For the classification of DLBCL subtypes as well as for as for providing a prognosis of the clinical outcome, the analysis of the tumor microenvironment and, particularly, of the different types and functions of tumor-associated macrophages, is indispensable. Until now, however, most information about macrophages is obtained either in a completely indirect way by gene expression profiling or by manual counts in immunohistochemically (IHC) fluorescence stained tissue samples while automated recognition of single IHC stained macrophages remains a difficult task. In an accompanying publication, a reliable approach to this problem has been established, and a large set of related images has been generated and analyzed.
Results: Provided image data comprise a) fluorescence microscopy images of 44 multiple immunohistostained DLBCL tumor subregions, captured at four channels corresponding to CD14, CD163, Pax5 and DAPI; b) "cartoon-like" TV-filtered versions of these images, generated by Rudin-Osher-Fatemi (ROF) denoising; c) an automatically generated mask of the evaluation subregion, based on information from the DAPI channel, and d) automatically generated segmentation masks for macrophages, B-cells and all cell nuclei, using information from CD14, CD163, Pax5 and DAPI channels, respectively.
Conclusions: A large set of IHC stained DLBCL specimens is provided together with segmentation masks for different cell populations generated by a reference method for automated image analysis, thus featuring considerable reuse potential.

Key words: lymphoma, DLBCL, macrophage, multiple immunohistochemical staining, automated cell counting, ROF filtering, floating threshold, rule-based detection, image dataset

Compiled on: February 7, 2020.
Draft manuscript prepared by the author.

Data Description

Context

We present an image dataset generated as a part of an accompanying publication, which is concerned with method development and comparison for automated segmentation and counting of macrophages in diffuse large B-cell lymphoma (DLBCL) tissue sections [1]. DLBCL is an aggressive cancer disease which is characterized by a large heterogeneity of pathological, clinical and biological features [2]. Therefore, a crucial step for the classification of DLBCL subtypes as well as for providing a prognosis of the clinical outcome is the analysis of the tumor microenvironment in terms of counts, local distributions and functions of the different cell populations and, particularly, of the tumor-associated macrophages occuring there [3].

Until now, most information about macrophages is obtained either by gene expression profiling [4] or by manual counts in immunohistochemically (IHC) stained tissue microarrays or high-power fields, thus either gathering information in a completely indirect way or accepting extreme subsampling rates [5]. A reliable approach for fully automated segmentation, identification and counting of IHC stained macrophages within whole tissue slides has been addressed in [1].

Our dataset contains monochrome fluorescence microscopy images of 44 DLBCL tissue samples wherein different macrophage populations (using antibodies against CD14 and CD163) and B-cells (using antibody against Pax5) as well as all cell nuclei (using DAPI) have been stained and imaged at different wavelengths. Further, we supply processed images, comprising "cartoon-like" TV-filtered images (generated by Rudin-Osher-Fatemi filtering) as well as results of the automated macrophage segmentation. For this publication, we completed these data by automated segmentation of B-cells and the cell nuclei.

Methods

a) Preparation and staining of DLBCL tissue. From the files of the Lymph Node Registry Kiel, 44 DLBCL biopsy specimens have been selected. For every specimen, from formalin-fixed paraffin-embedded tissue a slice of 2 μm thickness has been obtained. In order to detect specific macrophages and its relation to B-cells, a triple IHC staining has been done, using primary antibodies against CD14 (Cell Marque, Cat# 114R-14, RRID: AB_2827391; 1:10), CD163 (Novus, Cat# NB110-59935, RRID: AB_892323; 1:100) and Pax5 (Santa Cruz Biotechnology, Cat# sc-1974, RRID: AB_2159678; 1:100) labelled with donkey anti rabbit Alexa 488, donkey anti mouse Alexa 555 and donkey anti goat Alexa 647 (all from Invitrogen, Thermo Fisher Scientific, Waltham, MA, USA; 1:100) as secondary antibodies. Subsequently, the slices have been incubated with DAPI (Invitrogen, Thermo Fisher Scientific, Waltham, MA, USA; 1:5000) and cover-slipped with mounting medium. Use of tissue was in accordance with the guidelines of the internal review board of the Medical Faculty of the Christian-Albrechts-University Kiel, Germany (No. 447/10).

b) Selection of tumor subregions and image acquisition. Within every tissue sample, the tumor area was defined and marked by a pathologist based on inspection of conventional Haematoxylin-Eosin (HE) staining in a neighboring reference slice. Subsequently, within the IHC stained slice, a rectangular subregion of the tumor area has been selected, taking care for acceptable tissue and staining quality. Maximum size of tumor subregions is 10 mm^2.

Images of tumor subregions within the IHC stained slides have been captured by Hamamatsu Nanozoomer 2.0 RS slide scanner (Hamamatsu Photonics, Ammersee, Germany) with $20\times$ magnification at four wavelengths, resulting in single images for the CD14, CD163, Pax5 and DAPI channels, respectively, which were saved in `.ndpi` output format with default settings as used in clinical trial routine. Note that, at this point, moderate built-in compression by imaging device was accepted. Single-channel raw images have been converted into `.tif` format without further compression and sliced into tiles of 1000×1000 px format (at right and lower border, the sizes may be smaller), using the software package ImageJ with the extension ndpitools [6]. The resulting monochrome images have been further converted from RGB into greyscale mode using the modulus of the RGB vector and finally saved in losslessly compressed `.png` format. We refer to them as to images of type "original". Let us remark that image acquisition and tiling have been performed in such a way that no spatial misalignment between the scans at the different wavelengths occurred. Pixel size is $0.45\times0.45\ \mu\mathrm{m}^2$ in all images.

c) Image processing. For every tile, the segmentation method from [1] has been applied to the CD14, CD163, Pax5 and DAPI channel images, resulting in ROF-filtered images (saved as type "cartoon"), a mask for the evaluation subregion within the tile, indicating the presence of tissue at all, as inferred from DAPI channel information (saved as type "evalmask"), and segmentations of macrophages within the CD14 and CD163 channels (saved as type "segment"). Due to the large inhomogeneity of IHC staining, even across a single target macrophage, we provide two further masks containing the convex hulls of the segmented features instead of the features themselves (saved as type "convhull"). The segmentation masks for double-stained macrophages are saved as type "multiple". For a general description of the ROF filter based segmentation method, we refer to [1]. Here, we describe in more detail the generation of segmentations for the Pax5 and DAPI channels, which are new in this paper.

Let us recall the notation from [1] where the indices i and j count the current intensity threshold and the features to be inspected at this stage, $s(F_j)$, $c(F_j)$ and $r(F_j)$ denote the size of a feature F_j itself, the size of its convex hull and the ratio of the principal axes' lengths of the smallest ellipse covering the feature, respectively. s_{min}, s_{max}, c_{max} and r_{max} denote the minimal and maximal feature size (in px), the maximal area excess of the convex hull (in percent) and the maximal ratio of axes, respectively.

In order to obtain a segmentation of the DAPI channel, the ROF-filtered image has been further subjected to a local Narendra-Fitch contrast enhancement [7]

$$p(k,l)_{enhanced} = m(k,l) + \frac{c}{\sigma(k,l)}\cdot\Big(p(k,l)_{original} - m(k,l)\Big) \quad (1)$$

where $c>0$ is a weight parameter and $m(k,l)$, $\sigma(k,l)$ denote the mean and standard deviation of the intensities within a subregion centered at the pixel $p(k,l)_{original}$, respectively. We used $c = 0.75$ and a square subregion of 11×11 px size. Then, in a first run, Steps 3 – 10 of the ROF filter based segmentation have been applied, using the bounds $s_{min} = 60$ and $s_{max} = 119$ for the feature size but modifying geometrical rule No. 3) for feature classification from [1] as follows: If $s_{min} \le s(F) \le s_{max}$ then test whether the feature satisfies both of the criteria 3b) $r(F_j) \le r_{max}$ (the feature is not too elongated) and 3d) $c(F_j)/s(F_j) \le 1 + c_{max}/100$ (the deviation from circular shape is bounded from above). If yes, save the feature F_j into the output mask, interpreting it as a cell nucleus, and mask it in $I^{(3)}(i)$. If not then neglect the feature and mask it in $I^{(3)}(i)$ as well. Here, we used the parameter values $r_{max} = 2.5$ and $c_{max} = 150$. In a second run, Steps 3 – 10 of the ROF filter based segmentation have been repeated with the parameter settings $s_{min} = 120$ and

s_{max} = 180, using again the described modification of rule No. 3) but saving only those features into the output mask which are completely disjoint to the output of the first run. Finally, the results of both runs have been combined into a single mask (saved as type "segment"). Within a further result mask of type "convhull", the convex hulls of the detected features have been stored.

For the segmentation of the B-cells, the ROF-filtered image of the Pax5 channel has been subjected to a moderate Narendra-Fitch contrast enhancement as well, using the parameter c = 0.1 and a square subregion of 15×15 px size. To the result, Steps 3 – 10 of the ROF filter based segmentation have been applied, using the bounds s_{min} = 80 and s_{max} = 159 as well as the described modification of rule No. 3) with parameters r_{max} = 2.5 and c_{max} = 150 but saving into the output mask (of type "segment") only features which intersection with the convex hull of some cell nucleus, as obtained in the segmentation of the DAPI channel, is nonempty. Thus, numerous artifacts appearing in the Pax5 staining will be excluded. Again, the convex hulls of the dectected features have been stored within a further mask of type "convhull".

Dataset structure

Image data are organized by tissue specimens (top-level folders) and tiles (second-level folders), the latter ones ordered by position. Top-level folders are named `specimen_01`, ... , `specimen_44`; second-level folders are named e.g. `specimen_01_tile_01_01`, ... , `specimen_01_tile_09_08`. Within each second-level folder, 19 image files in greyscale ("gs") or black-and-white ("bw") mode are stored in losslessly compressed `.png` format with 8-bit or 1-bit depth, respectively. Table 1 and Figure 1 summarize the different images available at a given tile. The filenames are built as `specimen_xx_tile_yy_zz_channel_[CD14, CD163, Pax5, DAPI]_type_[original, cartoon, segment, convhull, multiple, evalmask]_mode_[gs, bw].png`. Size of losslessly compressed `.png` image files has been minimized by application of OptiPNG routine [8]. Moreover, a logfile named `specimen_xx_tile_yy_zz__logfile.txt` is provided, containing detailed information about procedures, parameters and results of automated segmentation.

Table 1. Image files available within a given second-level folder.

Channel	Description	Type	Mode
CD14	CD14 staining, original single-channel image	original	gs
	ROF filtered image derived from original	cartoon	gs
	mask highlighting the segmented macrophages	segment	bw
	mask highlighting the convex hulls of the segmented macrophages	convhull	bw
	mask highlighting the segmented macrophages bearing CD163 staining as well	multiple	bw
CD163	CD163 staining, original single-channel image	original	gs
	ROF filtered image derived from original	cartoon	gs
	mask highlighting the segmented macrophages	segment	bw
	mask highlighting the convex hulls of the segmented macrophages	convhull	bw
	mask highlighting the segmented macrophages bearing CD14 staining as well	multiple	bw
Pax5	Pax5 staining, original single-channel image	original	gs
	ROF filtered image derived from original	cartoon	gs
	mask highlighting the segmented B-cells	segment	bw
	mask highlighting the convex hulls of the segmented B-cells	convhull	bw
DAPI	DAPI staining, original single-channel image	original	gs
	ROF filtered image derived from original	cartoon	gs
	mask representing the evaluation subregion	evalmask	bw
	mask highlighting the segmented cell nuclei	segment	bw
	mask highlighting the convex hulls of the segmented cell nuclei	convhull	bw

d) BLC2 scoring. For all specimens, a BCL2 score is available, see Table 2. It is based on BCL2-staining for tissue slides obtained from the same biopsy specimens as before but not necessarily adjacent to the slides used for the generation of the image data presented here. Staining was microscopically examined and semi-quantitatively scored by an experienced pathologist. Each stained slide was evaluated for the percentage of stained tumor cells by visual estimation in a representative tumor area. The estimated value was graded into following scores: 0 — all cells negative, 1 — up to 25% positive cells, 2 — 25% – 50% positive cells, 3 — 50% – 75% positive cells, 4 — over 75% positive cells.

Reuse potential

Although there is a vast number of publications concerned with the composition of tumor microenvironment in various types of lymphoma disease, image datasets of IHC stained cancer tissue are rarely publicly accessible if at all, cf. the discussion in [9]. Most data generated for the purpose of such analyses are not findable or not even accessible. For example, the Genomic Data Commons Data Portal of the National Cancer Institute [10, 11] currently lists only 48 cases of mature B-cell lymphoma with an image of a HE-stained slide available, while IHC stainings are missing at all. In this situation, the image

dataset presented in this note constitutes a document of interest in itself.

We will outline the most important options for further use of the data. First, it allows for a detailed morphometrical investigation of the imaged macrophages and B-cells with respect to the distribution of geometrical parameters as size, diameter, perimeter, etc., as well as to overall shape patterns. Second, the data may be used for validation, calibration and comparison of cell segmentation methods (manual, automated) and related software packages, making available a large reference dataset together with the output of a reference method as described in [1]. Note that, for these purposes, it is particularly adequate to use data admitting a routine quality level. Third, the original images as well as the segmentations presented here could be used for the generation of a sufficiently large training set for automated macrophage detection by machine learning methods. Fourth, the data may be used for study of co-localization and clustering of macrophages and B-cells within lymphoma tissue and cancer microenvironment, employing appropriate methods of point-pattern statistics [12, 13]. Finally, the dataset enables a closer study of the double-stained macrophage subpopulation. In order to facilitate a possible further processing of the obtained features (e.g. extraction of barycenters, replacement of the features by equally sized circles or squares), not only the masks for the segmented features themselves but as well for its convex hulls are provided.

To illustrate the described reuse potential, we include a set of composite figures, each combining information from several separate images. Figure 2.A shows an original image at CD14 channel (greyscale, original contrast-enhanced by factor 3.5 and inverted) with superimposition of the mask of the evaluation subregion, as obtained from the DAPI channel (light blue), and the segmentation of the CD14-stained macrophages (olive green). Figure 2.B shows the same tile as imaged at the Pax5 channel (greyscale, original inverted) with superimposition of the cell nuclei segmentation from DAPI channel (light blue, convex hulls) and the segmentation of the CD163-stained macrophages (dark yellow). In Figure 2.C, for the same tile, both macrophage segmentations (olive green or dark yellow, convex hulls) are combined in order to reveal double-stained parts (light yellow). In Figure 2.D, we superimposed to Figure 2.C the segmentation of B-cells from the Pax5 channel (magenta and grey, convex hulls). Observe that in Figs. 2.B and 2.D, some B-cells are positioned inside of macrophages, indicating that they are engulfed by the macrophages for phagocytosis (examples marked by arrows). It is obvious that co-localization and clustering patterns as empirically noticeable here must be investigated on a sound base of statistical methodology.

To improve reusability, BLC2 scores for the biopsy specimens are provided.

Table 2. BLC2 scores.

Score	Specimen No.
0	03, 05, 18, 32, 37, 43, 44
1	—
2	36
3	28
4	01, 02, 04, 06 – 17, 19 – 27, 29 – 31, 33 – 35, 38 – 42

Availability of supporting data

All image data are made publicly accessible under CC0 1.0 license at the Leipzig Health Atlas (LHA) repository [14] and can be reached from the address [15]. Each top-level folder can be downloaded as `.zip` file and bears a separate identifier, e.g. `https://health-atlas.de/lha/7YXMMFNPDG-0` within the repository, see Table 3. Two folders with total size larger than 1 GB (Nos. 04 and 44) have been splitted into a pair of files. Snapshots of the datasets are available in the GigaScience GigaDB repository as well [16].

Table 3. Datasets available at the Leipzig Health Atlas.

Name	Size (MB)	Identifier
specimen_01.zip	161	7YXMMFNPDG-0
specimen_02.zip	142	7YXXYUTPYN-9
specimen_03.zip	121	7YXY2MUWDK-3
specimen_04a.zip	630	7YXYECRXQM-0
specimen_04b.zip	709	7YXYY60JX7-9
specimen_05.zip	653	7YY08G00A0-4
specimen_06.zip	168	7YY0NHJXF8-2
specimen_07.zip	409	7YY0X073KU-7
specimen_08.zip	396	7YY146X8HE-4
specimen_09.zip	283	7YY19AWR7C-8
specimen_10.zip	368	8004FF6QR6-5
specimen_11.zip	708	8004RQHWHX-6
specimen_12.zip	360	800516PXMC-9
specimen_13.zip	150	8005NDPDNX-6
specimen_14.zip	283	8005QXY7QG-0
specimen_15.zip	124	8005TY4388-4
specimen_16.zip	146	8005X2U355-5
specimen_17.zip	364	80062H7C7J-4
specimen_18.zip	164	800HNVWTJX-5
specimen_19.zip	105	800HR9GPJE-5
specimen_20.zip	418	800J5AN4V1-7
specimen_21.zip	431	800JDAJXHV-6
specimen_22.zip	465	802X2RAVTV-8
specimen_23.zip	333	802X8VYQ27-0
specimen_24.zip	462	802XR67DWU-2
specimen_25.zip	749	802Y1JFKPQ-6
specimen_26.zip	635	803AHC5EAH-5
specimen_27.zip	137	803AHW6TD9-4
specimen_28.zip	225	803AU2NYKJ-8
specimen_29.zip	549	803C11PMP7-3
specimen_30.zip	334	803C4Q94NP-5
specimen_31.zip	217	803C809ERJ-6
specimen_32.zip	293	803CFU4J96-9
specimen_33.zip	330	803CJR62YA-8
specimen_34.zip	474	803NYKMOPY-9
specimen_35.zip	286	803PH07HQT-2
specimen_36.zip	225	803PJKT2JG-7
specimen_37.zip	563	803PKWG9XG-9
specimen_38.zip	524	803PPV4R44-8
specimen_39.zip	879	8044GTGCPG-1
specimen_40.zip	382	8044J0U5JC-0
specimen_41.zip	421	804GHX9A2E-8
specimen_42.zip	114	804GJF4HQ4-8
specimen_43.zip	596	804GY21PMN-9
specimen_44a.zip	506	804H6EM8W2-5
specimen_44b.zip	452	804H7C4T1P-0

Declarations

List of abbreviations

DAPI: 4',6-diamidino-2-phenylindole; DLBCL: diffuse large B-cell lymphoma; HE: Haematoxylin-Eosin; IHC: immunohistochemical(ly); LHA: Leipzig Health Atlas; ROF: Rudin-Osher-Fatemi; TV: total variation.

Ethical Approval

Tissue usage is covered by statement No. 447/10 of the internal review board of the Medical Faculty of the Christian-Albrechts-University Kiel, Germany.

Consent for publication

Not applicable.

Competing Interests

The authors declare that they have no competing interests.

Funding

SR, RH and MW have been funded by BMBF project "MMML-Demonstrators", grants no. 031A428C and 031A428D.

Author's Contributions

MW performed the image processing and wrote the manuscript. SR performed the IHF staining and image generation. RH curated the large-size image datasets and managed the storage within the Leipzig Health Atlas repository. WK identified the cohort. UDB contributed to the Context, Methods and Reuse potential sections. All authors read and approved the final manuscript.

Acknowledgements

The authors would like to thank Dana Germer and Charlotte Botz-von Drathen for their excellent technical support.

References

1. Wagner M, Hänsel R, Reinke S, Richter J, Altenbuchinger M, Braumann UD, Spang R, Löffler M, Klapper W. Automated macrophage counting in DLBCL tissue samples: a ROF filter based approach. Biol Proc Online 21 (2019) : 13 (electronically published)
2. Swerdlow SH, Campo E, Harris NL, Jaffe ES, Pileri SA, Stein H, Thiele J, Vardiman, JW (Eds). WHO Classification of Tumours of Haematopoietic and Lymphoid Tissues. WHO Classification of Tumours, Vol. 2. International Agency for Research on Cancer; Lyon 2017. 4th, rev. ed.
3. Scott DW, Gascoyne RD. The tumour microenvironment in B cell lymphomas. Nat Rev Cancer 14 (2014) : 517 – 534
4. Scott DW, Wright GW, Williams PM, Lih CJ, Walsh W, Jaffe ES, Rosenwald A, Campo E, Chan WC, Connors JM, Smeland EB, Mottok A, Braziel RM, Ott G, Delabie J, Tubbs RR, Cook JR, Weisenburger DD, Greiner TC, Glinsmann-Gibson BJ, Fu K, Staudt LM, Gascoyne RD, Rimsza LM. Determining cell-of-origin subtypes of diffuse large B-cell lymphoma using gene expression in formalin-fixed paraffin embedded tissue. Blood 123 (2014) : 1214 – 1217
5. Lozanski G, Pennell M, Shana'ah A, Zhao W, Gewirtz A, Racke F, Hsi E, Simpson S, Mosse C, Alam S, Swierczynski S, Hasserjian RP, Gurcan MN. Inter-reader variability in follicular lymphoma grading: conventional and digital reading. J Pathol Inform 4 (2013) : 30
6. Deroulers C, Ameisen D, Badoual M, Gerin C, Granier A, Lartaud M. Analyzing huge pathology images with open source software. Diagnostic Pathology 8 (2013) : 92
7. Narendra PM, Fitch RC. Real-time adaptive contrast enhancement. IEEE Trans Pattern Analysis Machine Int 3 (1981) : 655 – 661
8. `http://optipng.sourceforge.net` (accessed 06.02.2020)
9. Kostopoulos S, Ravazoula P, Asvestas P, Kalatzis I, Xenogiannopoulos G, Cavouras D, Glotsos D. Development of a reference image collection library for histopathology image processing, analysis and decision support systems research. J Digit Imaging 30 (2017) : 287 – 295
10. `https://portal.gdc.cancer.gov/repository` (accessed 06.02.2020)
11. Cooper LAD, Demicco EG, Saltz JH, Powell RT, Rao A, Lazar AJ. PanCancer insights from The Cancer Genome Atlas: the pathologist's perspective. J Pathol 244 (2018) : 512 – 524
12. Ripley BD. Spatial Statistics. Wiley; New York 1981
13. Møller J, Waagepetersen RP. Modern statistics for spatial point processes. Scand J Statistics 34 (2007) : 643 – 684
14. Meineke FA, Löbe M, Stäubert S. Introducing technical aspects of research data management in the Leipzig Health Atlas. Stud Health Technol Inform 247 (2018) : 426 – 430
15. `https://health-atlas.de/lha/7XWCUQPR8K-8` (accessed 06.02.2020)
16. Wagner M, Reinke S, Hänsel R, Klapper W, Braumann UD. Supporting data for "An image dataset related to automated macrophage detection in immunostained lymphoma tissue samples". GigaScience Database (2020), `http://dx.doi.org/10.5524/100703` (accessed 06.02.2020)

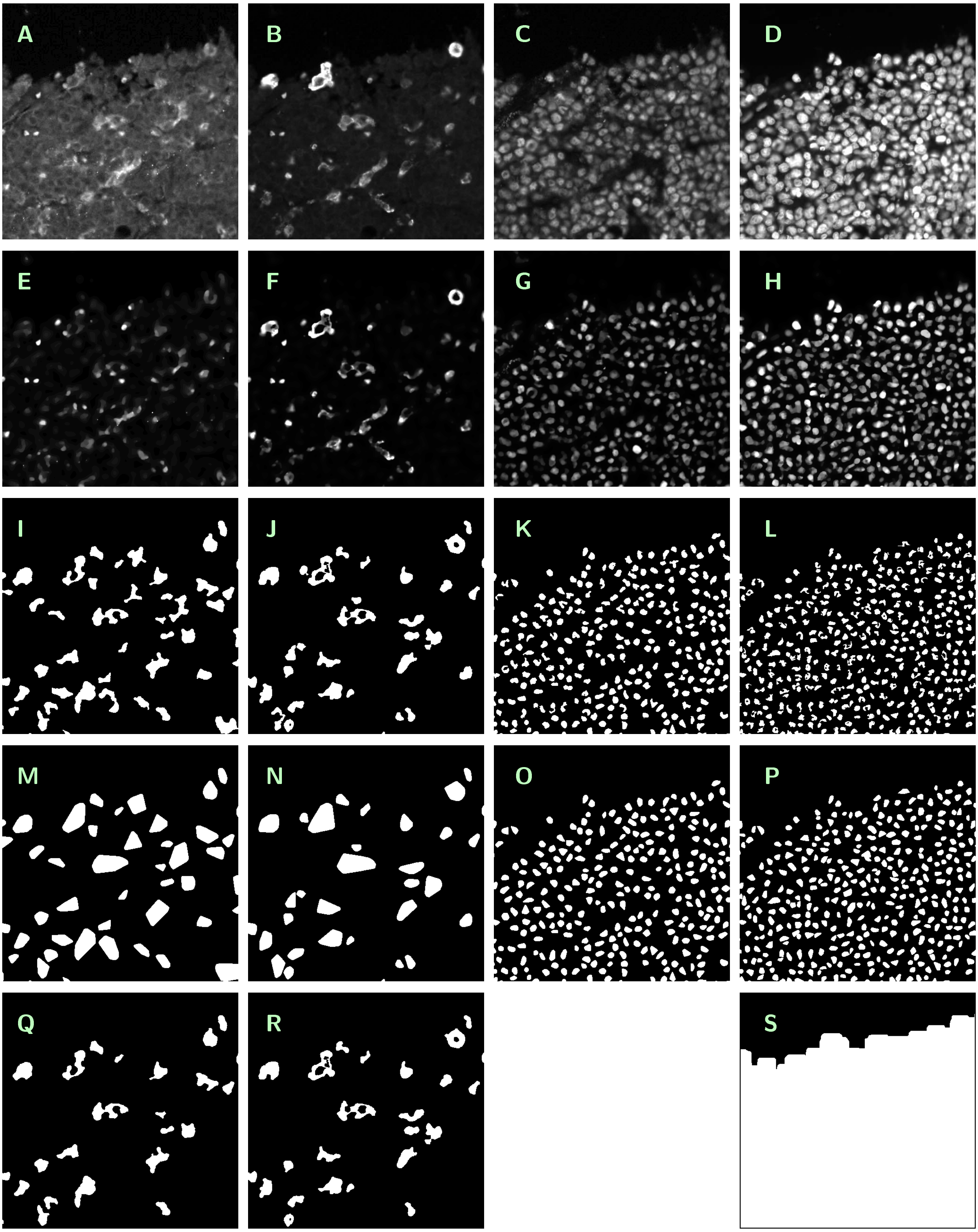

Figure 1. Summary of images available at a given tile (cutouts of 500 × 500 px size). Contrast enhanced in A by factor 3.5, in E by factor 7 and in F, G and H by factor 2.
Originals (A–D). A — specimen_02_tile_01_06_channel_CD14_type_original_mode_gs.png, B — specimen_02_tile_01_06_channel_CD163_type_original_mode_gs.png, C — specimen_02_tile_01_06_channel_Pax5_type_original_mode_gs.png, D — specimen_02_tile_01_06_channel_DAPI_type_original_mode_gs.png,
Cartoons (E–H). E — specimen_02_tile_01_06_channel_CD14_type_cartoon_mode_gs.png, F — specimen_02_tile_01_06_channel_CD163_type_cartoon_mode_gs.png, G — specimen_02_tile_01_06_channel_Pax5_type_cartoon_mode_gs.png, H — specimen_02_tile_01_06_channel_DAPI_type_cartoon_mode_gs.png,
Segmentations (I–L). I — specimen_02_tile_01_06_channel_CD14_type_segment_mode_bw.png, J — specimen_02_tile_01_06_channel_CD163_type_segment_mode_bw.png, K — specimen_02_tile_01_06_channel_Pax5_type_segment_mode_bw.png, L — specimen_02_tile_01_06_channel_DAPI_type_segment_mode_bw.png,
Convex hulls (M–P). M — specimen_02_tile_01_06_channel_CD14_type_convhull_mode_bw.png, N — specimen_02_tile_01_06_channel_CD163_type_convhull_mode_bw.png, O — specimen_02_tile_01_06_channel_Pax5_type_convhull_mode_bw.png, P — specimen_02_tile_01_06_channel_DAPI_type_convhull_mode_bw.png,
Various (Q–S). Q — specimen_02_tile_01_06_channel_CD14_type_multiple_mode_bw.png, R — specimen_02_tile_01_06_channel_CD163_type_multiple_mode_bw.png, S — specimen_02_tile_01_06_channel_DAPI_type_evalmask_mode_bw.png.

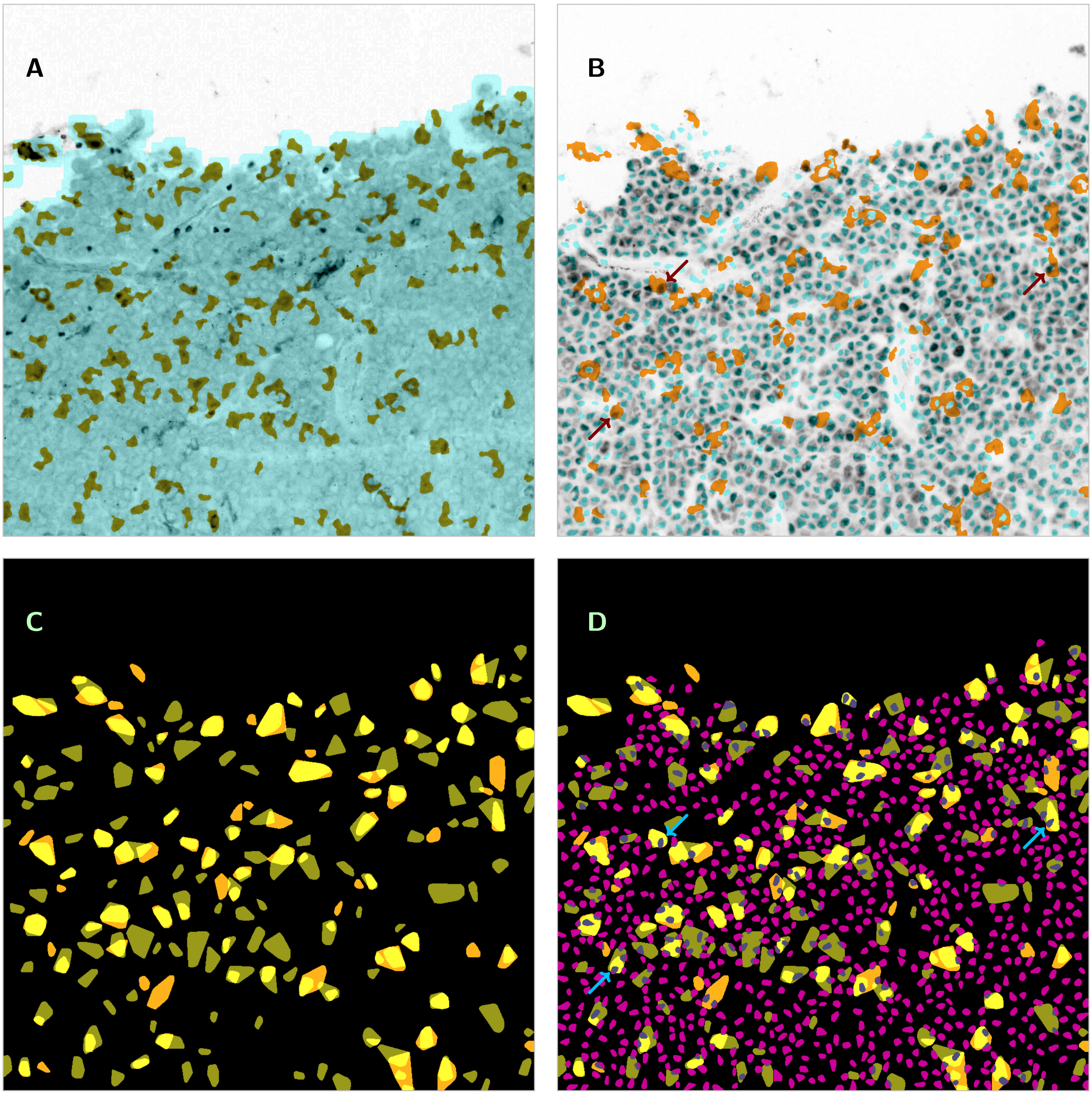

Figure 2. Examples of combined information from several images, based on `specimen_02_tile_01_06_...` Image size is 1000 × 1000 px (450 × 450 μm^2).
A — Original image at CD14 channel (greyscale, contrast enhanced by factor 3.5, inverted) (`channel_CD14_type_original_mode_gs.png`) with superimposition of the mask of the evaluation subregion, as obtained from the DAPI channel (light blue) (`channel_DAPI_type_evalmask_mode_bw.png`) and the segmentation of the CD14-stained macrophages (olive green) (`channel_CD14_type_segment_mode_bw.png`).
B — The same tile as imaged at the Pax5 channel (greyscale, inverted) (`channel_Pax5_type_original_mode_gs.png`) with superimposition of the cell nuclei segmentation from DAPI channel (light blue, convex hulls) (`channel_DAPI_type_convhull_mode_bw.png`) and the segmentation of the CD163-stained macrophages (dark yellow) (`channel_CD163_type_segment_mode_bw.png`). Examples of B-cells positioned inside of macrophages indicated by arrows.
C — Combination of both macrophage segmentations (olive green or dark yellow, convex hulls) for the same tile in order to reveal double-stained parts (light yellow) (`channel_CD14_type_convhull_mode_bw.png` / `channel_CD163_type_convhull_mode_bw.png`).
D — Segmentation of B-cells from the Pax5 channel (magenta and grey, convex hulls) (`channel_Pax5_type_convhull_mode_bw.png`) superimposed to Figure 2.C. Examples of B-cells positioned inside of macrophages indicated by arrows (the same cells as in Figure 2.B).

Comments to the reviewers
Click here to access/download;Personal Cover;comments_to_reviewers.pdf

GigaScience

Submitted paper No. GIGA-D-19-00311 -- 1st revision

"An image dataset related to automated macrophage detection in immunostained lymphoma tissue samples"

Authors: Marcus Wagner; Sarah Reinke; René Hänsel; Wolfram Klapper; Ulf-Dietrich Braumann

Answers to Reviewer's comments

The authors would like to thank both reviewers to valuable comments, which helped us to improve the quality of the paper.

Reviewer #1: *This Data Note, entitled "An image dataset related to automated macrophage detection in immunostained lymphoma tissue samples", is of great interest from a clinical perspective. Macrophages are known to orchestrate the local immune response via cytokine expression, and are additionally critical mediators of tissue remodelling through secretion of macrophage elastase and matrix metalloproteinases. In addition, macrophage activation, which is observed in chronic inflammation, has been used as a model system to understand chromatin remodelling. In this study, the authors outline a means of detecting tumour-associated macrophages in diffuse large B-cell lymphoma (DLBCL) tissue sections.*

The manuscript is well written, and the segmentation process used to generate masks is briefly outlined in the Methods. The total data volume for this dataset is 18GB and these are provided as multiple zip files. I reviewed a subset of these zip files and the data was very well organised with a grayscale image file (raw data) and 8-bit black and white images that represent segmentation masks for each of the four channels (CD14, CD163, Pax5, DAPI). The image data is of cellular-resolution, with a pixel size of 0.45um x 0.45um. Furthermore, there is a metadata file for each tissue sample that details the maxima and minima for each of the fluorescent channels.

Due to the highly significant role of macrophages in health and disease, I think there is great biomedical interest in this dataset. The authors indeed stress the importance of this dataset with the following point in the Reuse potential section: "Most data generated for the purpose of such analyses are not findable or not even accessible. For example, the Genomic Data Commons Data Portal of the National Cancer Institute [10, 11] currently lists only 48 cases of mature B-cell lymphoma with an image of a HE-stained slide available, while IHC stainings are missing at all."

-- Remark: The number of documented cases at this site has not been increased per 09.12.2019.

I commend the authors for stressing this point and for making these image data publicly available as CC0.

Minor comments:

1. The authors have made the segmentation masks available as 8-bit grayscale images. However, as grayscale information is not utilised in these images, the masks could have been presented more simply as binary images that only show black and white. From a reuse perspective, could the authors explain if there is any additional benefit in making the segmentation masks available as 8-bit grayscale images?

-- No additional benefit. As a consequence, BW images within the datasets have been re-stored with 1-bit depth now.

2. The authors have additionally generated masks that contain the convex hulls of segmented macrophages, B-cells, and nuclei. In a previous publication, the authors state the importance of generating convex hulls for macrophage analysis with the following: "With regard to the possible nonuniformity of the staining of single macrophages, it is obvious that the distribution of the macrophage sizes should be observed from the convex hulls of the features rather than from the features themselves." (Wagner et al., Biol Proced Online. 2019; 21: 13.)

From a reuse perspective, I was wondering whether the authors could comment on whether there is additional value in using the convex hull to perform morphometry of Pax5-positive lymphoma cells? Likewise, is there any benefit that the authors can outline for using the convex hull for morphometry of DAPI-stained nuclei?

--There is a benefit even in the case of Pax5- and DAPI-stained features. As shown in Fig. 1, 3rd and 4th column, staining of these features is not so inhomogeneous as for the macrophages but still not completely homogeneous. Obtained segmentations reflect this fact. However, further processing steps as calculation of barycenters, replacement of features by circles of equal size etc. can be much more easily performed with convex hulls. This has been shortly remarked in Reuse section.

3. In Figure 2, the colours are not referred to in the figure legend, and rather the reader has to refer to the main body of the text. The figure legends should be sufficiently detailed that the reader does not have to refer to the main body of the text. Consequently, I would like the colours that are used in Figure 2 to be detailed in the figure legend.

-- Changed. The legend of Figure 2 is now completely self-explaining without reference to the main text body.

4. In Figure 2D, one observes the overlap between the convex hull of Pax5-positive B-cells (magenta), and the convex hull of CD163-positive macrophages (yellow). When referring to Figure 2D in the main body of the text (see section entitled "Reuse potential"), the authors state the following: "Observe that some B-cells are positioned inside of macrophages, indicating that they are engulfed by the macrophages for phagocytosis."

However, I think it is alternatively possible that this overlap does not represent phagocytosis, but rather is an artefact created by using convex hull operations. To ensure that this is not the case, can the authors provide an equivalent tiled image to that used

in Figure 2D, but which shows the overlap between: 1) the mask of segmented macrophages; and 2) the mask of segmented B-cells. I wish to compare this image - which does not use convex hulls - with Figure 2D so that I can be sure that the authors statement about B-cells being positioned inside macrophages is valid.

-- Overlap of macrophages and B-cells is not artificially created by convex hull generation. Compare Fig. 2. B, where an superimposition of the mask of segmented CD163+ macrophages and the original Pax5 staining is already shown. Even here, before forming of convex hulls, one observes that some heavily stained B-cells are positioned inside of the macrophages. In order to make examples of such cases unambiguously visible, some arrows were added in Figs. 2. B and 2. D.

5. The tumour microenvironment of diffuse large B-cell lymphomas (DLBCLs) is notoriously heterogeneous. I was wondering whether there are additional prognostic markers that the authors counted on their image data? For example, did the authors use morphological criteria to score apoptosis on DLBCL tissue sections? From a reuse perspective, differences in apoptotic index - if captured - would be particularly useful as it could be used for machine learning-based classification. On a related note, it would be additionally useful to know whether dysregulated Bcl-2 (B-cell lymphoma 2) family gene expression - which is associated with resistance to apoptosis in B-cell lymphomas - was observed in any of the samples in this set of 44 DLBCL tissue samples.

-- For all specimens, a semi-quantitative BLC2 score is available and has been provided now in Table 2. A short remark concerning BLC2 scoring has been included into the Methods section. Attribution of further biomarkers to the specimens is still under investigation and will be published in future.

Reviewer #2: *Review of 'An image dataset related to automated macrophage detection in immunostained lymphoma tissue samples' by Wagner, et al.*

This paper presents a dataset comprised of cancerous tissue samples which were stained for B-cells and macrophages, then imaged using fluorescence microscopy. The collected images are segmented and the masks resulting from these segmentations are presented along with the original image data.

I have a few minor comments about the paper.

The authors wrote: "Single channel raw images have been converted into uncompressed .tif format and sliced into tiles of 1000x1000 px format (at right and lower border, the sizes may be smaller), using the software package ImageJ with the extension ndpitools [6]. The resulting monochrome images have been further converted from RGB into greyscale mode using the modulus of the RGB vector and ¬finally saved in losslessly compressed .png format."

I am looking at the file "specimen_05_tile_01_01_channel_Pax5_type_original_mode_gs.png"

This image has compression artifacts which are both immediately noticeable and are also very severe.

-- Here was indeed a missing point in data description. Compression artifacts are present, and they were generated during the built-in initial storing within the imaging device. In the following processing steps, no further compresssion was generated.

However, the present image quality can be accepted for the following three reasons. 1) The settings used at the imaging device are default in clinical trial routine. 2) All analyses in Wagner et al. (2019) were based on the original images published here. 3) Data to be used for validation and comparison of cell segmentation methods should admit a routine quality level.

These points have been addressed in the Methods and Reuse sections of the revised paper.

The authors use the phrase "the total of cell nuclei" to indicate the results of a DAPI staining. This is not the correct phrasing. The authors use the word "cartoon" to describe the output of an image processing operation. Cartoon is not the right word here. The authors use the abbreviation "resp." This abbreviation cannot be used in formal writing.

-- Reformulated in all cases.

ORCID data of authors

Marcus Wagner: 0000-0001-9063-1452

Sarah Reinke: 0000-0001-9729-4030

René Hänsel: 0000-0001-8344-0658

Wolfram Klapper: 0000-0001-7208-4117

Ulf-Dietrich Braumann: 0000-0002-0987-4498
