# Supplementary material for: An image dataset related to automated macrophage detection in immunostained lymphoma tissue samples
Source: Gigascience. 2020 Mar 12;9(3):giaa016. doi: 10.1093/gigascience/giaa016 (PMC7066390; doi:10.1093/gigascience/giaa016)
Supplement: giaa016_Response_to_Reviewer_Comments_Original_Submission [file giaa016_response_to_reviewer_comments_original_submission.pdf]

Author's Response To Reviewer Comments

Close

GigaScience

Submitted paper No. GIGA-D-19-00311 -- 1st revision

"An image dataset related to automated macrophage detection in immunostained lymphoma tissue samples"

Authors: Marcus Wagner; Sarah Reinke; René Hänsel; Wolfram Klapper; Ulf-Dietrich Braumann

Answers to Reviewer's comments

The authors would like to thank both reviewers to valuable comments, which helped us to improve the quality of the paper.

Reviewer #1: " (...) The authors indeed stress the importance of this dataset with the following point in the Reuse potential section: "Most data generated for the purpose of such analyses are not findable or not even accessible. For example, the Genomic Data Commons Data Portal of the National Cancer Institute [10, 11] currently lists only 48 cases of mature B-cell lymphoma with an image of a HE-stained slide available, while IHC stainings are missing at all."

-- Remark: The number of documented cases at this site has not been increased per 09.12.2019.

"I commend the authors for stressing this point and for making these image data publicly available as CC0.

Minor comments:

1. The authors have made the segmentation masks available as 8-bit grayscale images. However, as grayscale information is not utilised in these images, the masks could have been presented more simply as binary images that only show black and white. From a reuse perspective, could the authors explain if there is any additional benefit in making the segmentation masks available as 8-bit grayscale images?"

-- No additional benefit. As a consequence, BW images within the datasets have been re-stored with 1-bit depth now.

"2. The authors have additionally generated masks that contain the convex hulls of segmented macrophages, B-cells, and nuclei. In a previous publication, the authors state the importance of generating convex hulls for macrophage analysis with the following: "With regard to the possible nonuniformity of the staining of single macrophages, it is obvious that the distribution of the macrophage sizes should be observed from the convex hulls of the features rather than from the features themselves." (Wagner et al., Biol Proced Online. 2019; 21: 13.) From a reuse perspective, I was wondering whether the authors could comment on whether there is additional value in using the convex hull to perform morphometry of Pax5-positive lymphoma cells? Likewise, is there any benefit that the authors can outline for using the convex hull for morphometry of DAPI-stained nuclei?"

--There is a benefit even in the case of Pax5- and DAPI-stained features. As shown in Fig. 1, 3rd and 4th column, staining of these features is not so inhomogeneous as for the macrophages but still not completely homogeneous. Obtained segmentations reflect this fact. However, further processing steps as calculation of barycenters, replacement of features by circles of equal size etc. can be much more easily performed with convex hulls. This has been shortly remarked in Reuse section.

"3. In Figure 2, the colours are not referred to in the figure legend, and rather the reader has to refer to the main body of the text. The figure legends should be sufficiently detailed that the reader does not

have to refer to the main body of the text. Consequently, I would like the colours that are used in Figure 2 to be detailed in the figure legend."

-- Changed. The legend of Figure 2 is now completely self-explaining without reference to the main text body.

"4. In Figure 2D, one observes the overlap between the convex hull of Pax5-positive B-cells (magenta), and the convex hull of CD163-positive macrophages (yellow). When referring to Figure 2D in the main body of the text (see section entitled "Reuse potential"), the authors state the following: "Observe that some B-cells are positioned inside of macrophages, indicating that they are engulfed by the macrophages for phagocytosis." However, I think it is alternatively possible that this overlap does not represent phagocytosis, but rather is an artefact created by using convex hull operations. To ensure that this is not the case, can the authors provide an equivalent tiled image to that used in Figure 2D, but which shows the overlap between: 1) the mask of segmented macrophages; and 2) the mask of segmented B-cells. I wish to compare this image - which does not use convex hulls - with Figure 2D so that I can be sure that the authors statement about B-cells being positioned inside macrophages is valid."

-- Overlap of macrophages and B-cells is not artificially created by convex hull generation. Compare Fig. 2. B, where an superimposition of the mask of segmented CD163+ macrophages and the original Pax5 staining is already shown. Even here, before forming of convex hulls, one observes that some heavily stained B-cells are positioned inside of the macrophages. In order to make examples of such cases unambiguously visible, some arrows were added in Figs. 2. B and 2. D.

"5. The tumour microenvironment of diffuse large B-cell lymphomas (DLBCLs) is notoriously heterogeneous. I was wondering whether there are additional prognostic markers that the authors counted on their image data? For example, did the authors use morphological criteria to score apoptosis on DLBCL tissue sections? From a reuse perspective, differences in apoptotic index - if captured - would be particularly useful as it could be used for machine learning-based classification. On a related note, it would be additionally useful to know whether dysregulated Bcl-2 (B-cell lymphoma 2) family gene expression - which is associated with resistance to apoptosis in B-cell lymphomas - was observed in any of the samples in this set of 44 DLBCL tissue samples."

-- For all specimens, a semi-quantitative BLC2 score is available and has been provided now in Table 2. A short remark concerning BLC2 scoring has been included into the Methods section. Attribution of further biomarkers to the specimens is still under investigation and will be published in future.

Reviewer #2: "Review of 'An image dataset related to automated macrophage detection in immunostained lymphoma tissue samples' by Wagner, et al. This paper presents a dataset comprised of cancerous tissue samples which were stained for B-cells and macrophages, then imaged using fluorescence microscopy. The collected images are segmented and the masks resulting from these segmentations are presented along with the original image data.

I have a few minor comments about the paper.
The authors wrote: "Single channel raw images have been converted into uncompressed .tif format and sliced into tiles of 1000x1000 px format (at right and lower border, the sizes may be smaller), using the software package ImageJ with the extension ndpitools [6]. The resulting monochrome images have been further converted from RGB into greyscale mode using the modulus of the RGB vector and finally saved in losslessly compressed .png format." I am looking at the file "specimen_05_tile_01_01_channel_Pax5_type_original_mode_gs.png" This image has compression artifacts which are both immediately noticeable and are also very severe."

-- Here was indeed a missing point in data description. Compression artifacts are present, and they were generated during the built-in initial storing within the imaging device. In the following processing steps, no further compresssion was generated.

However, the present image quality can be accepted for the following three reasons. 1) The settings used at the imaging device are default in clinical trial routine. 2) All analyses in Wagner et al. (2019) were based on the original images published here. 3) Data to be used for validation and comparison of cell segmentation methods should admit a routine quality level.

These points have been addressed in the Methods and Reuse sections of the revised paper.

"The authors use the phrase "the total of cell nuclei" to indicate the results of a DAPI staining. This is not the correct phrasing. The authors use the word "cartoon" to describe the output of an image processing operation. Cartoon is not the right word here. The authors use the abbreviation "resp." This abbreviation cannot be used in formal writing."

-- Reformulated in all cases.

Close
