# Supplementary material for: An image dataset related to automated macrophage detection in immunostained lymphoma tissue samples
Source: Gigascience. 2020 Mar 12;9(3):giaa016. doi: 10.1093/gigascience/giaa016 (PMC7066390; doi:10.1093/gigascience/giaa016)
Supplement: giaa016_Response_to_Reviewer_Comments_Revision_1 [file giaa016_response_to_reviewer_comments_revision_1.pdf]

Author's Response To Reviewer Comments

Close

The required changes and insertions have been made. Thank you for your assistance with RRIDs. ORCIDs of all authors are included in a separate file as part of the submission.

Close
