# Supplementary material for: An image dataset related to automated macrophage detection in immunostained lymphoma tissue samples
Source: Gigascience. 2020 Mar 12;9(3):giaa016. doi: 10.1093/gigascience/giaa016 (PMC7066390; doi:10.1093/gigascience/giaa016)
Supplement: giaa016_Reviewer_1_Report_Original_Submission — Chris Armit -- 9/23/2019 Reviewed [file giaa016_reviewer_1_report_original_submission.pdf]

Reviewer Report

Title: An image dataset related to automated macrophage detection in immunostained lymphoma tissue samples

Version: Original Submission **Date:** 9/23/2019

Reviewer name: Chris Armit

Reviewer Comments to Author:

This Data Note, entitled "An image dataset related to automated macrophage detection in immunostained lymphoma tissue samples", is of great interest from a clinical perspective. Macrophages are known to orchestrate the local immune response via cytokine expression, and are additionally critical mediators of tissue remodelling through secretion of macrophage elastase and matrix metalloproteinases. In addition, macrophage activation, which is observed in chronic inflammation, has been used as a model system to understand chromatin remodelling. In this study, the authors outline a means of detecting tumour-associated macrophages in diffuse large B-cell lymphoma (DLBCL) tissue sections.

The manuscript is well written, and the segmentation process used to generate masks is briefly outlined in the Methods. The total data volume for this dataset is 18GB and these are provided as multiple zip files. I reviewed a subset of these zip files and the data was very well organised with a grayscale image file (raw data) and 8-bit black and white images that represent segmentation masks for each of the four channels (CD14, CD163, Pax5, DAPI). The image data is of cellular-resolution, with a pixel size of 0.45um x 0.45um. Furthermore, there is a metadata file for each tissue sample that details the maxima and minima for each of the fluorescent channels.

Due to the highly significant role of macrophages in health and disease, I think there is great biomedical interest in this dataset. The authors indeed stress the importance of this dataset with the following point in the Reuse potential section:

"Most data generated for the purpose of such analyses are not findable or not even accessible. For example, the Genomic Data Commons Data Portal of the National Cancer Institute [10, 11] currently lists only 48 cases of mature B-cell lymphoma with an image of a HE-stained slide available, while IHC stainings are missing at all."

I commend the authors for stressing this point and for making these image data publicly available as CC0.

Minor comments

1. The authors have made the segmentation masks available as 8-bit grayscale images. However, as grayscale information is not utilised in these images, the masks could have been presented more simply as binary images that only show black and white. From a reuse perspective, could the authors explain if there is any additional benefit in making the segmentation masks available as 8-bit grayscale images?

2. The authors have additionally generated masks that contain the convex hulls of segmented macrophages, B-cells, and nuclei. In a previous publication, the authors state the importance of generating convex hulls for macrophage analysis with the following:

"With regard to the possible nonuniformity of the staining of single macrophages, it is obvious that the distribution of the macrophage sizes should be observed from the convex hulls of the features rather than from the features themselves." (Wagner et al., Biol Proced Online. 2019; 21: 13.)
From a reuse perspective, I was wondering whether the authors could comment on whether there is additional value in using the convex hull to perform morphometry of Pax5-positive lymphoma cells? Likewise, is there any benefit that the authors can outline for using the convex hull for morphometry of DAPI-stained nuclei?
3. In Figure 2, the colours are not referred to in the figure legend, and rather the reader has to refer to the main body of the text. The figure legends should be sufficiently detailed that the reader does not have to refer to the main body of the text. Consequently, I would like the colours that are used in Figure 2 to be detailed in the figure legend.
4. In Figure 2D, one observes the overlap between the convex hull of Pax5-positive B-cells (magenta), and the convex hull of CD163-positive macrophages (yellow). When referring to Figure 2D in the main body of the text (see section entitled "Reuse potential"), the authors state the following:
"Observe that some B-cells are positioned inside of macrophages, indicating that they are engulfed by the macrophages for phagocytosis."
However, I think it is alternatively possible that this overlap does not represent phagocytosis, but rather is an artefact created by using convex hull operations. To ensure that this is not the case, can the authors provide an equivalent tiled image to that used in Figure 2D, but which shows the overlap between: 1) the mask of segmented macrophages; and 2) the mask of segmented B-cells. I wish to compare this image - which does not use convex hulls - with Figure 2D so that I can be sure that the authors statement about B-cells being positioned inside macrophages is valid.
5. The tumour microenvironment of diffuse large B-cell lymphomas (DLBCLs) is notoriously heterogeneous. I was wondering whether there are additional prognostic markers that the authors counted on their image data? For example, did the authors use morphological criteria to score apoptosis on DLBCL tissue sections? From a reuse perspective, differences in apoptotic index - if captured - would be particularly useful as it could be used for machine learning-based classification. On a related note, it would be additionally useful to know whether dysregulated Bcl-2 (B-cell lymphoma 2) family gene expression - which is associated with resistance to apoptosis in B-cell lymphomas - was observed in any of the samples in this set of 44 DLBCL tissue samples.

Level of Interest

Please indicate how interesting you found the manuscript: Choose an item.

Quality of Written English

Please indicate the quality of language in the manuscript: Choose an item.

Declaration of Competing Interests

Please complete a declaration of competing interests, considering the following questions:

- Have you in the past five years received reimbursements, fees, funding, or salary from an organisation that may in any way gain or lose financially from the publication of this manuscript, either now or in the future?
- Do you hold any stocks or shares in an organisation that may in any way gain or lose financially from the publication of this manuscript, either now or in the future?
- Do you hold or are you currently applying for any patents relating to the content of the manuscript?
- Have you received reimbursements, fees, funding, or salary from an organization that holds or has applied for patents relating to the content of the manuscript?
- Do you have any other financial competing interests?
- Do you have any non-financial competing interests in relation to this paper?

If you can answer no to all of the above, write 'I declare that I have no competing interests' below. If your reply is yes to any, please give details below.

I declare that I have no competing interests

I agree to the open peer review policy of the journal. I understand that my name will be included on my report to the authors and, if the manuscript is accepted for publication, my named report including any attachments I upload will be posted on the website along with the authors' responses. I agree for my report to be made available under an Open Access Creative Commons CC-BY license (http://creativecommons.org/licenses/by/4.0/). I understand that any comments which I do not wish to be included in my named report can be included as confidential comments to the editors, which will not be published.

Choose an item.

To further support our reviewers, we have joined with Publons, where you can gain additional credit to further highlight your hard work (see: https://publons.com/journal/530/gigascience). On publication of this paper, your review will be automatically added to Publons, you can then choose whether or not to claim your Publons credit. I understand this statement.

Yes Choose an item.
