# Supplementary material for: An image dataset related to automated macrophage detection in immunostained lymphoma tissue samples
Source: Gigascience. 2020 Mar 12;9(3):giaa016. doi: 10.1093/gigascience/giaa016 (PMC7066390; doi:10.1093/gigascience/giaa016)
Supplement: giaa016_Reviewer_2_Report_Original_Submission — Guy M Hagen, PHD -- 9/26/2019 Reviewed [file giaa016_reviewer_2_report_original_submission.pdf]

Reviewer Report

Title: An image dataset related to automated macrophage detection in immunostained lymphoma tissue samples

Version: Original Submission **Date:** 9/26/2019

Reviewer name: Guy M Hagen, PHD

Reviewer Comments to Author:

Review of 'An image dataset related to automated macrophage detection in immunostained lymphoma tissue samples' by Wagner, et al.

This paper presents a dataset comprised of cancerous tissue samples which were stained for B-cells and macrophages, then imaged using fluorescence microscopy. The collected images are segmented and the masks resulting from these segmentations are presented along with the original image data.

I have a few minor comments about the paper.

The authors wrote: "Single channel raw images have been converted into uncompressed .tif format and sliced into tiles of 1000x1000 px format (at right and lower border, the sizes may be smaller), using the software package ImageJ with the extension ndpitools [6]. The resulting monochrome images have been further converted from RGB into greyscale mode using the modulus of the RGB vector and Â¬finally saved in losslessly compressed .png format."

I am looking at the file "specimen_05_tile_01_01_channel_Pax5_type_original_mode_gs.png"

This image has compression artifacts which are both immediately noticeable and are also very severe.

The authors use the phrase "the total of cell nuclei" to indicate the results of a DAPI staining. This is not the correct phrasing. The authors use the word "cartoon" to describe the output of an image processing operation. Cartoon is not the right word here. The authors use the abbreviation "resp." This abbreviation cannot be used in formal writing.

Level of Interest

Please indicate how interesting you found the manuscript: Choose an item.

Quality of Written English

Please indicate the quality of language in the manuscript: Choose an item.

Declaration of Competing Interests

Please complete a declaration of competing interests, considering the following questions:

- Have you in the past five years received reimbursements, fees, funding, or salary from an organisation that may in any way gain or lose financially from the publication of this manuscript, either now or in the future?
- Do you hold any stocks or shares in an organisation that may in any way gain or lose financially from the publication of this manuscript, either now or in the future?
- Do you hold or are you currently applying for any patents relating to the content of the manuscript?
- Have you received reimbursements, fees, funding, or salary from an organization that holds or has applied for patents relating to the content of the manuscript?
- Do you have any other financial competing interests?
- Do you have any non-financial competing interests in relation to this paper?

If you can answer no to all of the above, write 'I declare that I have no competing interests' below. If your reply is yes to any, please give details below.

I declare that I have no competing interests

I agree to the open peer review policy of the journal. I understand that my name will be included on my report to the authors and, if the manuscript is accepted for publication, my named report including any attachments I upload will be posted on the website along with the authors' responses. I agree for my report to be made available under an Open Access Creative Commons CC-BY license (http://creativecommons.org/licenses/by/4.0/). I understand that any comments which I do not wish to be included in my named report can be included as confidential comments to the editors, which will not be published.

Choose an item.

To further support our reviewers, we have joined with Publons, where you can gain additional credit to further highlight your hard work (see: https://publons.com/journal/530/gigascience). On publication of this paper, your review will be automatically added to Publons, you can then choose whether or not to claim your Publons credit. I understand this statement.

Yes Choose an item.
